# Influenza A H5N1 and H7N9 in China: A spatial risk analysis

**DOI:** 10.1371/journal.pone.0174980

**Published:** 2017-04-04

**Authors:** Chau Minh Bui, Lauren Gardner, Raina MacIntyre, Sahotra Sarkar

**Affiliations:** 1 School of Public Health and Community Medicine, University of New South Wales, Sydney, New South Wales, Australia; 2 School of Civil and Environmental Engineering, University of New South Wales, Sydney, New South Wales, Australia; 3 College of Public Service & Community Solutions, Arizona State University, Phoenix, Arizona, United States of America; 4 Departments of Philosophy and Integrative Biology, University of Texas, Austin, Texas, United States of America; Shanxi University, CHINA

## Abstract

**Background:**

Zoonotic avian influenza poses a major risk to China, and other parts of the world. H5N1 has remained endemic in China and globally for nearly two decades, and in 2013, a novel zoonotic influenza A subtype H7N9 emerged in China. This study aimed to improve upon our current understanding of the spreading mechanisms of H7N9 and H5N1 by generating spatial risk profiles for each of the two virus subtypes across mainland China.

**Methods and findings:**

In this study, we (i) developed a refined data set of H5N1 and H7N9 locations with consideration of animal/animal environment case data, as well as spatial accuracy and precision; (ii) used this data set along with environmental variables to build species distribution models (SDMs) for each virus subtype in high resolution spatial units of 1km^2^ cells using Maxent; (iii) developed a risk modelling framework which integrated the results from the SDMs with human and chicken population variables, which was done to quantify the risk of zoonotic transmission; and (iv) identified areas at high risk of H5N1 and H7N9 transmission. We produced high performing SDMs (6 of 8 models with AUC > 0.9) for both H5N1 and H7N9. In all our SDMs, H7N9 consistently showed higher AUC results compared to H5N1, suggesting H7N9 suitability could be better explained by environmental variables. For both subtypes, high risk areas were primarily located in south-eastern China, with H5N1 distributions found to be more diffuse and extending more inland compared to H7N9.

**Conclusions:**

We provide projections of our risk models to public health policy makers so that specific high risk areas can be targeted for control measures. We recommend comparing H5N1 and H7N9 prevalence rates and survivability in the natural environment to better understand the role of animal and environmental transmission in human infections.

## Introduction

A zoonotic avian influenza virus (AIV) of subtype H5N1 emerged in humans in Hong Kong in 1997. The virus has since spread across Asia, Africa and Europe, and has infected over 854 humans and caused over 450 deaths [1]. In 2013 a new subtype H7N9 emerged in humans in China and the human case count by December 2016 was over 795, with over 314 deaths [2]. Differences in the epidemiology of H7N9 and H5N1 have previously been described: human H5N1 cases report higher severity of disease [3] and higher levels of contact with sick or dead birds [4]; H7N9 is asymptomatic in birds [5] and found at lower prevalence rates in poultry [6]; and the spatial distribution of H5N1 within a comparable time frame is considerably greater than that of H7N9 [3]. This study aimed to improve upon our current understanding of the spreading mechanisms behind H7N9 and H5N1, provide a geographic risk profile for each of the two virus subtypes across all of mainland China, and highlight the regions at greatest risk of experiencing AIV transmission.

Implementing effective AIV control measures in China to prevent spread among domestic poultry population, and transmission to humans, is a recognized challenge. Currently, there is growing government support for implementing fundamental changes in traditional poultry farming and marketing systems (such as centralised slaughtering of poultry, promotion of frozen poultry products, and increasing consumer awareness of disease risks) [7–9]. Implementing new province-wide regulation to poultry production and marketing systems is costly and logistically challenging. In contrast, implementing control measures in smaller, targeted areas allows for a more effective use of resources, easier management of adverse ramifications, and more specific assessments of interventions. A motivation for this study was to produce accurate risk assessment profiles at a spatially disaggregate scale, in order to identify the set of regions which should be targeted for control.

In this study we used species distribution models (SDMs) to develop geographic risk profiles for H5N1 and H7N9. SDMs are models which quantify the relationship between species distribution data (a set of locations representing sites where a species has been found), and a set of environmental covariates. SDMs can aid our understanding of virus spatial distributions, help to identify risk factors of disease occurrence, and reveal specific areas at highest risk of transmission which can be targeted for surveillance and control efforts. Examples of SDM algorithms include: Maxent, generalized linear models (GLMs), generalised additive models (GAMs), random forests or boosted regression trees (BRTs) [10]. SDMs are most commonly used in the fields of biogeography, conservation biology and ecology to identify environmental conditions which relate to species occurrence, estimate current species distribution and predict species distributions in new areas or under new environmental conditions [10,11]. In recent years, SDMs have been increasingly used in the public health context [12,13], particularly with respect to infectious diseases that involve vector species such as mosquito-borne diseases [14,15] and tick-borne diseases [16]; these diseases are markedly influenced by subtle changes in climatic conditions which determine vector distribution. In this study we created SDMs using Maxent for both H5N1 and H7N9.

A small set of previous studies have used SDMs to identify the set of high risk areas to target for control for both H5N1 [17–23] and H7N9 [24–30]. However, SDMs alone can only provide estimates of the probability of virus presence. In order to estimate the risk of disease transmission, a measure of interaction between animal and human hosts should additionally be considered. Thus, the risk assessment framework proposed in this study combined results from the generated SDMs with additional animal and human variables to capture such interaction. The risk methodology proposed in this work is similar to that described in Sarkar et al. [16] and Moffett et al. [15], which was developed for chagas disease and malaria, respectively. Similar to those works, we computed the relative risk of H5N1 or H7N9 human infection, defined as the risk in one cell compared to other cells in our study area. Within the context of AIVs, we found only four studies which have used a formal risk assessment method to build spatial risk maps. Spatial multi-criteria decision analysis (MCDA) methods were used in Stevens et al. [31] to develop risk maps for H5N1 in Asia, however the study only used imprecise coordinate data to validate their model. Paul et al. [32] improved on this risk model by applying the same methodology with improved coordinate data to develop H5N1 risk maps for Thailand and Cambodia. Hill et al. [33] developed a risk assessment framework to assess the risk of species jump, rather than risk of human infection. Li et al. [30] built SDMs individually for H5N1 and H7N9 using BRT methods and overlaid these with areas which have high genetic re-assortment potential (areas with high density of important animal hosts such as poultry, swine and wild birds) to identify areas where novel AIVs are likely to arise. These models were of low resolution (county level), and did not take into account location data precision and accuracy [30]. To our knowledge, no previous studies have applied a risk assessment framework to individually identify areas of high H5N1 and H7N9 risk, which conceptually differentiates the probability of virus presence in the environment regardless of being in or outside a host (i.e. SDM outputs) and combines it with a measure of zoonotic transmission efficiency (i.e. animal and human population densities) at the high level of spatial disaggregation provided in this work.

In this study we explicitly considered spatial precision and accuracy of coordinate data, and considered locations of positive virus detections in bird or bird-environments as positive virus locations. Producing SDMs using large spatial units can be affected by modifiable areal unit problems (MAUP) [34], while using centroid coordinates as location approximations can compromise the validity of a study [35]. Most previous H7N9 SDMs used aggregated data and low resolution spatial units, e.g. county-level analyses [26,27,30]–while few analysed data at high (e.g. approximately 1km^2^) resolutions [25,29]. Most SDM studies which built H5N1 models for China [18,36–39] used coordinate data from the World Organization for Animal Health (OIE), for which the accuracy of the case locations are not described [40]. Only Martin et al. [37] took accuracy of coordinate data into account. Furthermore, most H7N9 studies used only locations of positive human cases to define whether a location was infected [24–27,29,30]. Gilbert et al. [28] was the only H7N9 study to additionally consider locations of positive virus detections in bird or bird-environments as positive virus locations.

In summary, this study contributes to the literature by (i) collating a data set using case coordinates with a high level of accuracy that includes positive virus detections from humans, animals and animal environments; (ii) generating high-resolution (approximately 1km^2^ cells) SDMs for both H5N1 and H7N9, and (iii) performing high resolution risk analyses for both virus subtypes. The outcomes of this analyses were used to identify the areas at high risk for H5N1 and H7N9 transmission (both those which have previously reported cases and those which have not yet reported cases), which should be targeted for control measures.

## Materials and methods

### Study area

The whole of Mainland China was selected as the study area. Mainland China is defined as all regions under the direct jurisdiction of the People's Republic of China (PRC), excluding Hong Kong and Taiwan. All autochthonous H7N9 cases have so far been isolated from Mainland China, whereas Hong Kong and Taiwan have not yet reported autochthonous human or animal H7N9 cases.

The study area is approximately 9,596,960 km^2^, and is extremely diverse in terms of physical and human geography. Generally, the eastern and southern half of China consists of fertile, low-lying land and is more amenable to agricultural activity and human habitation, whereas the western and northern half of China largely consists of uninhabitable desserts, mountains and high plateaus, which are much less amenable to any anthropogenic activity.

All analyses were conducted in an unprojected, geographic coordinate system WGS84 (World Geodetic Survey 1984) using a spatial resolution of approximately 1km^2^ (0.0083 decimal degrees)–the study area is made up of 40 046 238 of these individual cells.

### Data

Disease occurrence data was collected from the EMPES-i georeferenced disease data repository compiled by the Food and Agricultural Organization (FAO) (http://empres-i.fao.org/eipws3g/, last accessed 21 September 2015). The Chinese Ministry of Agriculture avian influenza surveillance reports (www.syj.moa.gov.cn, last accessed September 2015) and official H7N9 World Organization of Animal Health (OIE) reports (www.oie.int, last accessed September 2015) were also used to extract the names of locations (e.g. LBMs, poultry farms, parks or wetlands) reported to have a positive sample of H5N1 or H7N9.

Maxent is designed to use only coordinate data which accurately reflect the true location of the case or outbreak. Hence, to maintain the integrity of our Maxent SDMs, we separated our disease occurrence data into two data sets: case records where the coordinates provided were associated with an acceptable level of accuracy and precision of at least 2 decimal places (to reflect a spatial resolution of approximately 1km^2^) were assigned to an “exact” data set; whereas case records with low or unknown location quality and low precision were assigned to an “unexact” data set.

EMPRES-i records included information on the locality, and an evaluation of the quality of location coordinates. All records where the precision of coordinates were labelled either administrative region centroids, or unknown were classified as “unexact”–only records where the precision of coordinates were specifically assigned “exact” were included in our “exact” data set.

For ministry and OIE records, latitude and longitude coordinate data were retrieved through searching location names in Chinese and English (using Baidu and Google mapping services, respectively). Coordinate data was assigned “exact” only when location names matched registered and georeferenced market (or farm/park) locations on Baidu and Google. Locations were matched by name, and address details when available (street name, district name, county name, city and province). Some location names provided by reports did not specify a distinctive name (only details such as district, county and province were provided)–these locations were not included in our exact data set.

Overall, we obtained coordinates for 267 H5N1 records, and 1289 H7N9 records. We obtained “exact” coordinates for 52 H5N1 records and 69 H7N9 records, and we have provided these data sets in a downloadable excel format (S1 and S2 Files). We summarised our H5N1 and H7N9 exact data sets by year of outbreak or human case onset, type of host the virus was isolated from (e.g. human, animal or environmental sample), and locality type (e.g. market, farm, village). These are listed in S1 and S2 Tables, and plotted in S1 and S2 Figs. We refer to each record as a ‘case’ throughout this paper.

Environmental variables for SDM construction were obtained from the WorldClim database (www.wordlclim.org, last accessed 12 October 2015) and are listed in Table 1. Each layer was available at a resolution of 30 arc-seconds (0.008333° x 0.008333°). Elevation data was downloaded from the Shuttle Radar Topography Mission (SRTM) 90m Digital Elevation Database v4.1 (www.cgiar-csi.org, last accessed 12 October 2015). This data was initially available at a resolution of 1km^2^. The data was used to derive the slope, aspect and composite topographic index using the Spatial Analyst extension of ArcMap 10.2, as described in Minnesota Department of Agriculture [41]. Topographical layers were resampled to align to the WorldClim layers.

**Table 1:** Environmental layers for Species Distribution Model (SDM) construction.

Variable name
^a^Annual Mean Temperature
^a^Mean Diurnal Range
Isothermality
Temperature Seasonality
^a^Max Temperature of Warmest Month
^a^Min Temperature of Coldest Month
Temperature Annual Range
Mean Temperature of Wettest Quarter
Mean Temperature of Driest Quarter
Mean Temperature of Warmest Quarter
Mean Temperature of Coldest Quarter
^a^Annual Precipitation
^a^Precipitation of Wettest Month
^a^Precipitation of Driest Month
Precipitation Seasonality
Precipitation of Wettest Quarter
Precipitation of Driest Quarter
Precipitation of Warmest Quarter
Precipitation of Coldest Quarter
^a^90m digital elevation v41
^a^Aspect
^a^Slope
^a^Compound Topographic Index (CTI)

^a^These 11 environmental variables were chosen to be included in the subset analysis

We obtained human population data from the LandScan (2014)^™^ High Resolution global Population Data Set (copyrighted by UT-Battelle, LLC, operator of Oak Ridge National Laboratory under Contract No. DE-AC05-00OR22725 with the United States Department of Energy), available from (http://web.ornl.gov/sci/landscan/), last accessed October 2015. This data was originally available in a resolution of 30 arc-seconds and was resampled to align to the extent of WorldClim layers. Values at each cell represent the average or ambient human population distribution.

Domestic chicken population data were obtained from Livestock Geo-Wiki (http://www.livestock.geo-wiki.org/, last accessed October 2015), and details on data set construction were provided in Robinson et al. [42]. The authors provided separate domestic chicken population rasters: (i) for chickens occupying intensive production systems (typically high density poultry holdings located nearby urban areas) and (ii) for chickens in extensive production systems (typically low density poultry farms which have locations amenable to prediction based on agricultural/land properties). Data was provided at a resolution of 1km^2^ and was resampled to align to the extent of WorldClim layers. Values for each cell represented the number of birds per km^2^. A summary of the data are provided in S3 Table.

LBM data were requested from authors of previously published SDMs [27,30]. Base maps of Chinese administrative regions (primary and secondary) were obtained from the GADM database of Global Administrative Areas (http://www.gadm.org/, last accessed October 2015).

### Species distribution model construction

The Maxent software package (version 3.3.3k, available from https://www.cs.princeton.edu/~schapire/maxent/) [43] was used to construct 8 SDMs. Maxent software [43] is primarily used in ecological sciences to model species distributions although the algorithm has previously been used in regional and global studies of avian influenza [17,18,20,22,23,25]. Maxent uses a machine learning algorithm to produce an estimate of the ecological suitability of a species using a set of environmental layers and a set of accurate case coordinates [43]. It does this by fitting a probability distribution to a set of cells in a study region—it finds the probability distribution of maximum entropy (i.e. the most spread out distribution) subject to a set of environmental constraints that represent the species distribution [43]. For each cell in the study area, a relative environmental suitability for species presence is calculated (referred to as suitability values). Maxent consistently produces relatively accurate results, even when only small sample sizes are available (generally considered as n <100) [44–46]. A major advantage of Maxent is that it only requires presence data, making this method more advantageous over other genetic algorithms and regression methods. Instead of prediction of presence or absence, Maxent estimates the relative environmental suitability for presence of a species, which also allow for finer estimates. Additionally, most publically available epidemiological data consist of records of human infections and animal outbreaks ascertained from passive surveillance, whilst systematic active surveillance using formal random sampling methodologies are not routinely performed with regards to AIVs—and details of negative cases or outbreaks (i.e. ‘controls’) are not made publically available.

All environmental layers for model construction are listed in Table 1. The environmental data was included because climate factors are known to influence AIV occurrence. Examples include modulating survival of AIV in the environment, and modifying animal host behaviour (e.g. seasonal migration of ducks and geese) or human behaviour, or host susceptibility to infection. Observational studies and spatial-temporal analyses have shown AIV infections display seasonality patterns in humans and animals [47,48], solar cycles for example have been associated with pandemic influenza activity [49] and animal transmission studies of human influenza viruses have shown that changes in temperature and humidity affect transmission potential and virus binding [50]. Previous studies have also shown decreasing or increasing relative humidity influences virus stability (discussed in [50]). Ambient temperature can impact virus transmission due to physiological changes in the hosts which increase susceptibility to infections [51], for example, in wild bird species which carry LPAI AIVs transmission can increase dramatically during migratory seasons, as birds congregate in large numbers in water bodies, and their immunity levels are likely to be low due to the physical exertion required during migration. In addition, during breeding seasons the number of immunological naïve birds increases substantially around this time, thus increasing the amounts of AIV shed in the population [52].

Temperature and precipitation can influence survivability of AIVs in the environment: H5N1 can remain infectious for 1 day at 37°C, 5 days at 24°C and 8 weeks at 4°C in dry and wet faeces, and laboratory experiments have shown H7N9 virus stability changes with varying temperatures [53]. Also, weather variations may influence human activities—for example more people go to the markets on a sunny day, which may increase virus transmission [54].

Topographical variables (elevation, slope, aspect and composite topographic index) were also used for model construction. Topographical variables, particularly elevation and slope, are commonly used in ENMs, and have been included in previous AIV models [55]. The reasons for their inclusion were (i) they are widely available for most countries, and (ii) in some studies, they have been found to contribute significantly to AIV occurrence, possibly as a surrogate indicator for some unknown variable related to AIV risk [56]. Elevation is thought to relate to land coverage—for example, areas of high elevation are dominated by forests, whilst flat areas (such as plains, deltas and coastal regions) are most likely be used for agriculture or urban development [56]. Slope and aspect may similarly influence AIV distribution through vegetation and land coverage. CTI is a measure for the tendency of water to pool, and may indicate areas suitable for rice cropping or duck farming which have been associated with H5N1 occurrence in Asia [57]. A discussion of CTI’s influence on H5N1 transmission in Vietnam is provided in Saksena et al. [58]. Aspect values indicate the compass direction which slope faces, and can be derived from slope values [21,59].

A variety of landcover and satellite data sets exist, and have been used in previous SDM models [20,22,23,25,60]. The reasoning behind their use is similar to the reasoning behind use of climate variables (they are able to capture seasonality and thus waterfowl migration patterns). For our model, landcover variables and satellite data were not used as our SDMs should already account for landcover and seasonality as they were created using precipitation, temperature and topological variables.

Maxent was run using Auto features. The test:training ratio was set to 40:60 as recommended by Phillips and Dudík [43]. Logistic output formats were selected as recommended in Phillips and Dudík [43]. In logistic output formats, each cell is assigned a probability (a value between 0 and 1) which can be interpreted as the estimate of the probability of species presence or as relative suitability. No thresholds were selected. Prevalence was set to 0.5 (default value) as we were unable to obtain reliable H5N1 and H7N9 prevalence or detection rates for all of mainland China at high resolution. For H7N9, there is more uncertainty around prevalence rates as this virus is asymptomatic in birds [3]. For each study, 100 models were created and relevant outputs were averaged.

For each virus subtype we created 4 different Maxent models which varied by number of variables and study area (model parameter settings remained the same): Models 1–2 were built using the full set of environmental variables and the full study area; models 3–4 were built using a subset of variables and the full study area; models 5–6 were built using the full set of variables and a subset of the study area; models 7–8 were built using a subset of variables and a subset of the study area.

For models 3,4,7 and 8, which included a subset of the environmental variables, 11 of the 23 environmental variables were finally chosen to be included in the subset (similar to Sarkar et al. [16]): Annual mean temperature, mean diurnal range, maximum temperature of warmest month, minimum temperature of coldest month, annual precipitation, precipitation of wettest month, precipitation of driest month, plus the four topological variables (see Table 1).

For models 5–8, which were created using only a subset of the original geographic study area, we selected only primary administrative regions (provinces, municipalities, autonomous regions) which had at least one H5N1 or H7N9 case (see S3 Fig), with the following exceptions: (i) the provinces of Shaanxi, Sichuan and the Chongqing municipality don’t contain any H5N1 and H7N9 cases from our exact data set however we include these provinces as they are geographically contiguous with selected provinces; (ii) Xinjiang Uygur autonomous region covers an expansive area, however contained only one H7N9 case and was hence excluded from the analysis. In total, 22 of 31 primary administrative regions were selected as the subset study area.

We evaluated each of the 8 models based on the receiver operating characteristic (ROC) and area under the curve (AUC) values. The ROC plot is a plot of sensitivity and 1–specificity [61]. In Maxent, sensitivity refers to how correctly the model has predicted presence, and specificity corresponds to a measure of correctly predicted absences. A model with good fit will produce a ROC curve which maximises sensitivity for low values for 1 –specificity or false positive rate. The AUC quantifies the significance of the ROC, with AUC values of 0.5 indicating the model is no better than random, and a value of 1.0 indicating perfect fit. For each model, we averaged the testing and training AUC, and standard deviation of testing AUC, over 100 replicates.

Overfitting can be an issue with maximum entropy methods, particularly when data is sparse, and can result in distributions clustering around the case data. The Maxent software has a relaxation component (regularization) which is designed to counteract correlations. Overfitting is more likely to occur when large numbers of environmental layers are used due to correlations between variables. A sign of overfitting is when a model performs very well (i.e. has high AUC results on the training data set), however performs poorly for unseen data (i.e. for the test data set). We adopt the same methodology as Sarkar et al. [16] to test whether models built with the full set of environmental variables (n = 23), were suffering from overfitting. First, the difference between test and training AUC scores was calculated for each set of 100 replicate models. Normality was tested using the Shapiro test and as not all data were normally distributed, the non-parametric Mann-Whitney-Wilcoxon test was used to decide whether the distribution of AUC differences of models with using all variables (n = 23), compared to models with fewer variables (n = 11), were identical. All statistical computations were performed using R.

The way environmental factors drive AIV occurrence differs according to region [22,23]. Having such a large study area (i.e. the whole of mainland China) means there is a large selection of background points, and as there are only a sparse number of disease cases this may influence reliability of predictions [62–64]. If case data only fall into a subset of the study area, Phillips [63] recommends a solution is to only draw background points from this subset to improve model performance. Models 5–8 were developed using a subset of the original study area of mainland China, and compared to models 1–4 in terms of differences in distributions, AUC and overfitting.

### Risk model construction

SDMs alone can only provide estimates of the probability of virus presence. In order to estimate the likelihood of zoonotic transmission, a measure of transmission efficiency must be accounted for. As described in Hill et al. [33], for zoonotic transmission to take place, a susceptible human must be within range of an infected animal (or an animal environment contaminated by high viral shedding). The level of opportunity for human exposure from the virus is proportional to the product of the *number of infected animals* and the *number of susceptible humans* [65]. To estimate the risk of circulating viruses to cause human infection, the SDM outputs for H5N1 and H7N9 are combined with human and animal population density. We use domestic chickens as the representative animal host, as these animals make up the highest proportion of China’s poultry sector [66], are the most commonly identified animal host of H5N1 and H7N9 [67], and virus shedding occurs at a higher rate in chickens compared to other avian species [5].

We modify a formal risk assessment methodology described in Sarkar et al. [16]. Risk models were constructed for each virus subtype independently, whereby a value between 0 and 1 was computed for each cell representing the relative risk of a human infection of H5N1 or H7N9 compared to other cells. These models combine ecological factors with demographic and agricultural factors known to modulate AIV transmission, and disregard variations of risk from human interventions such as those described in the introduction (animal biosecurity, vaccination, LBM closures etc).

For each virus subtype, we use a simple multiplicative model for computing the risk, *r*_*k*_, for each cell *k* in the study area as shown in Eq (1).

$$r_{k=\;}p_{k}\cdot h_{k}\cdot c_{k}$$ (1)

This equation provides a measure of the risk posed to humans in cell *k* in regards to the likelihood of becoming infected with H5N1 or H7N9. The variable, *p*_*k*_, is the estimated prevalence of H5N1 or H7N9 in cell *k* (relative to other cells in the landscape) from the Maxent output. The variables, *h*_*k*_, and *c*_*k*_, represent human and chicken population, respectively, in cell *k*. Computed *r*_*k*_ values were normalised, as shown in Eq (2), by dividing by the highest value computed over all cells, *k*, in the study area. The final result, *R*_*k*_, is the relative risk of a human infection of H5N1 or H7N9 posed to each cell:

$$R_{k}=\;r_{k}/max_{k\;}\left(r_{k}\right)$$ (2)

A log transformation was used for the human population density and intensive and extensive chicken population density variables, due to their highly skewed nature. Intensive and extensive chicken population densities were summed, and treated as a single variable, *c*_*k*_, in the model. Both human and chicken population densities were normalized to the highest value over all cells, *k*, in the study area. A frequency histogram showing the distribution of each variable for the set of exact case locations for each virus subtype is provided in S4 Fig.

Based on the exact case data (S4 Fig) the majority of cases for both H5N1 and H7N9 fell in regions with medium human density and medium poultry densities, and less cases fell in low and high density regions. For chicken density this is possibly because H5N1 is more likely to occur in chicken farms with poor hygiene and biosecurity practices, regardless of the size or density of the farm. There is extreme variation in terms of biosecurity, hygiene and farming practices among chicken farms in Asia—and such variation is difficult to account for using the chicken density data sets that are currently available [55]. Stevens et al. [31] addressed this limitation by assuming a quadratic relationship between H5N1 risk and chicken density, with the highest risk areas being those with a medium density of chickens (between 500–5000 heads/km^2^). Their reasoning was based on a review of the literature [55], which found that most large commercial chicken farms (where chicken densities can be very high) often implement appropriate biosecurity regulations and are expected to have little risk of H5N1 occurrence. In contrast, medium-sized poultry farms (representing semi-commercial and backyard producers) are often the ones that are unregulated and have less stringent infection control. Subsistence farmers with very small flock densities are considered to have negligible risk due to insufficient hosts to sustain an outbreak. For human density the explanation is less obvious. One possibility is to do with the changing food consumption practices, which are more evident in urban areas—a literature review of food consumption practices in China found that people are moving away from the traditional practice of purchasing fresh market produce and preparing meals at home, to now eating outside of the home (e.g. in restaurants) and eating processed foods [68]. Hence, we assume that transmission risk is lower in low human density areas and extremely high human density areas. There are however, no large scale surveys of consumer preference specifically for frozen or live poultry in China.

Given these observed relationships between the exact case locations and respective spatial variables, the human and chicken density variables were reclassified to a scale of 0 to 1 based on a simple Gaussian fuzzy membership function prior to being used in the risk function. This transformation is illustrated in Eq 3.

$$Gaussian\left(x;c,\sigma \right)=\;e^{-\frac{1\;}{2}{\left(\;\frac{x-c}{\sigma }\;\right)}^{2}}$$ (3)

Let *x* be the transformed (log and normalized) human or chicken population variable in cell *k*, and let the parameter, *c*, represent the ideal membership for the set and any values where *x* = *c* is assigned the value of 1. As *x* values move away from the midpoint, their membership to the set gradually decreases. When *x* values are too distant from the ideal definition, they are no longer considered to be in the set and are assigned zeroes. The parameter,σ, represents the spread or width of the Gaussian function (Eq 3). Values of *c* and σ were selected for each variable based on the distribution (i.e. mean and standard deviation) of each variables in the set of locations where exact H5N1 and H7N9 cases lie. For H5N1 we used *c* = 0.532 and σ = 0.183 for chicken density, and *c* = 0.619 and σ = 0.198 for human density. For H7N9 we used *c* = 0.601 and σ = 0.103 for chicken density, and *c* = 0.689 and σ = 0.146 for human density. All spatial analyses were performed in ArcMap 10.2 [41].

## Results

### Species distribution models

**Fig 1:**
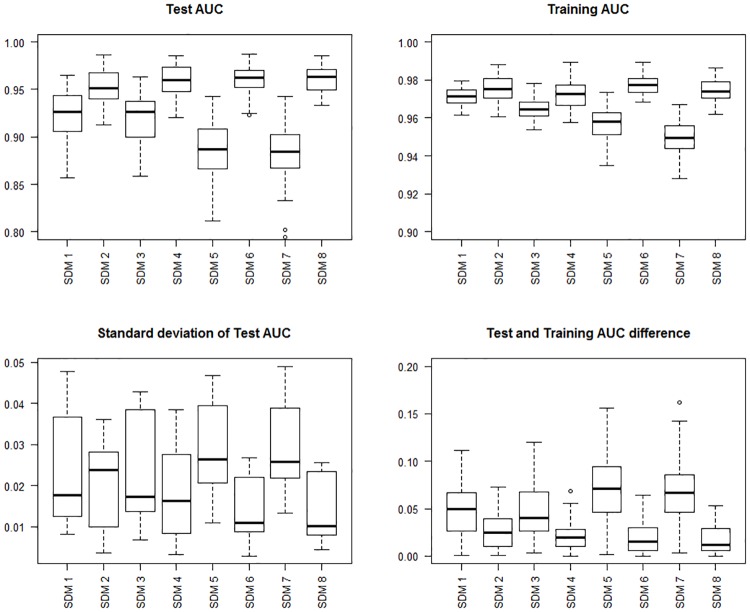
Species Distribution Model (SDMs) evaluation results. Top left panel shows boxplots of the area under the curve (AUC) for test data. The top right panel shows boxplots of AUC for training data. The bottom left panel shows boxplots of standard deviation of test data. The bottom right panel shows boxplots of test and training AUC differences.

**Fig 2:**
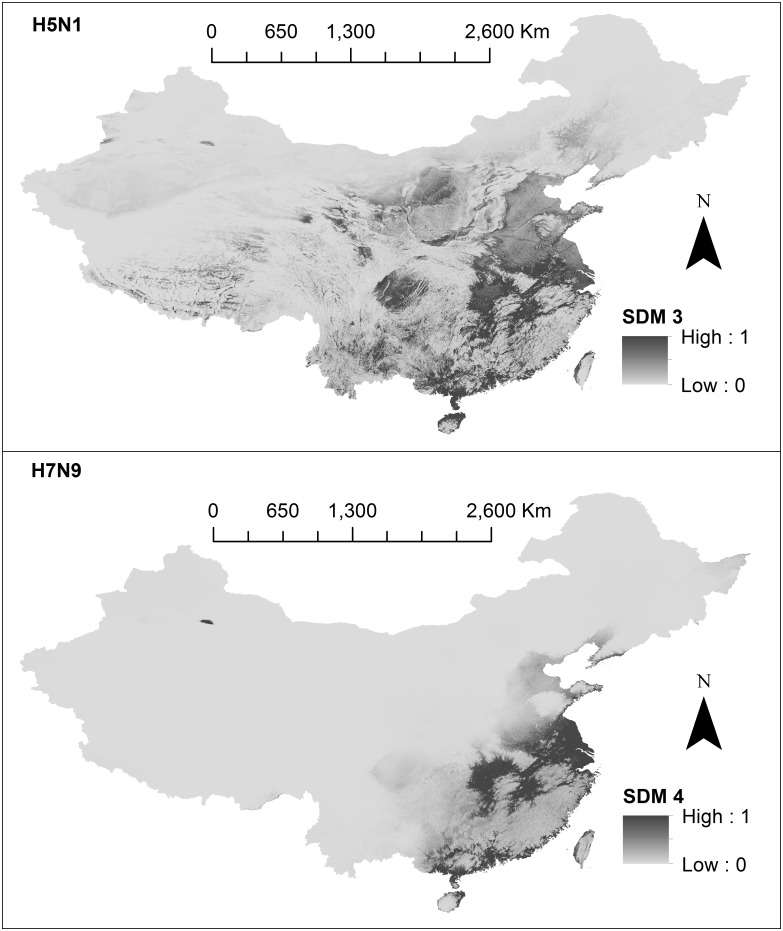
Species Distribution Models (SDMs) built using Maxent. The first panel shows H5N1 (SDM 3) and the second panel shows H7N9 (SDM 4). Suitability values for each cell (approximately 1km^2^) are represented on a continuous scale of low (light grey) to high (dark grey). SDMs were built using Maxent software version 3.3.3k (available from https://www.cs.princeton.edu/~schapire/maxent/). SDMs were developed using environmental variables, created using data from: the WorldClim database (www.wordlclim.org), the Shuttle Radar Topography Mission (SRTM) 90m Digital Elevation Database v4.1 (www.cgiar-csi.org). Data sources used to obtain the case locations to build SDMs include: the Food and Agricultural Organization (FAO) (http://empres-i.fao.org/eipws3g/), the Chinese Ministry of Agriculture Avian Influenza Surveillance Reports (www.syj.moa.gov.cn), the World Organization of Animal Health (OIE) reports (www.oie.int). Base maps were obtained from the GADM database of Global Administrative Areas (http://www.gadm.org/). Maps were built using ArcMap 10.2.

Based on model evaluation results (summarised in Table 2 and Fig 1), we chose SDMs 3 and 4 (see Fig 2) to be included in our risk model. Final SDMs 3 and 4 are shown in Fig 2, and the remaining models are shown in S5–S7 Figs. Prediction capacity was high for 6 of 8 models (AUC > 0.90). H7N9 SDM models (SDM 2,4,6 and 8) consistently performed better than H5N1 in terms of AUC. SDM models 1–2, suffered from overfitting based on Mann-Whitney-Wilcoxon tests (P-values turned out to be less than the significance level, hence the null hypothesis is rejected and AUC differences are non-identical). However overfitting was not an issue for SDMs 5–6. Reducing the study area (SDMs 5–8) did not make considerable differences in terms of suitability distribution (see S6 and S7 Figs), or AUC results (see Table 2). The term “suitability distribution” refers to the distribution of these suitability values in geographic space. For each subtype, the suitability for occurrence is displayed on a continuous scale from 0 (least suitable) to 1 (highly suitable). On visual appraisal, all 4 H5N1 suitability distributions were similar to each other; likewise, all 4 H7N9 suitability distributions were similar. H5N1 suitability distribution was distinctly more geographically diffuse across south-eastern China, whereas H7N9 suitability distribution was clustered densely around distinct areas (Zhejiang and Guangdong provinces). For H5N1, 5.8% (3 of 52) exact cases fell into low suitability areas (*p*_*k*_ <0.25), and for H7N9, 8.7% (6 of 69) exact cases fell into the low suitability areas (*p*_*k*_<0.25).

**Table 2:** Species Distribution Model (SDM) evaluation results.

	Model characteristics			Model evaluation results			
Model name	Subtype	Study area	Environmental variables	Mean AUC^a^ for Test data	Mean AUC^a^ for Training data	Mean standard deviation of Test AUC^a^	Wilcoxon rank sum test (overfitting)
SDM 1	H5N1	All	All	0.924	0.971	0.023	W = 7445, p < 0.001
SDM 2	H7N9	All	All	0.952	0.975	0.020	W = 8030.5, p < 0.001
SDM 3	H5N1	All	Subset	0.920	0.965	0.023	
SDM 4	H7N9	All	Subset	0.960	0.972	0.018	
SDM 5	H5N1	Subset	All	0.885	0.957	0.029	W = 5394, p = 0.336
SDM 6	H7N9	Subset	All	0.961	0.977	0.014	W = 5394, p = 0.336
SDM 7	H5N1	Subset	Subset	0.883	0.950	0.030	
SDM 8	H7N9	Subset	Subset	0.960	0.975	0.014	

^a^AUC refers to the area under the curve

### Risk analysis

**Fig 3:**
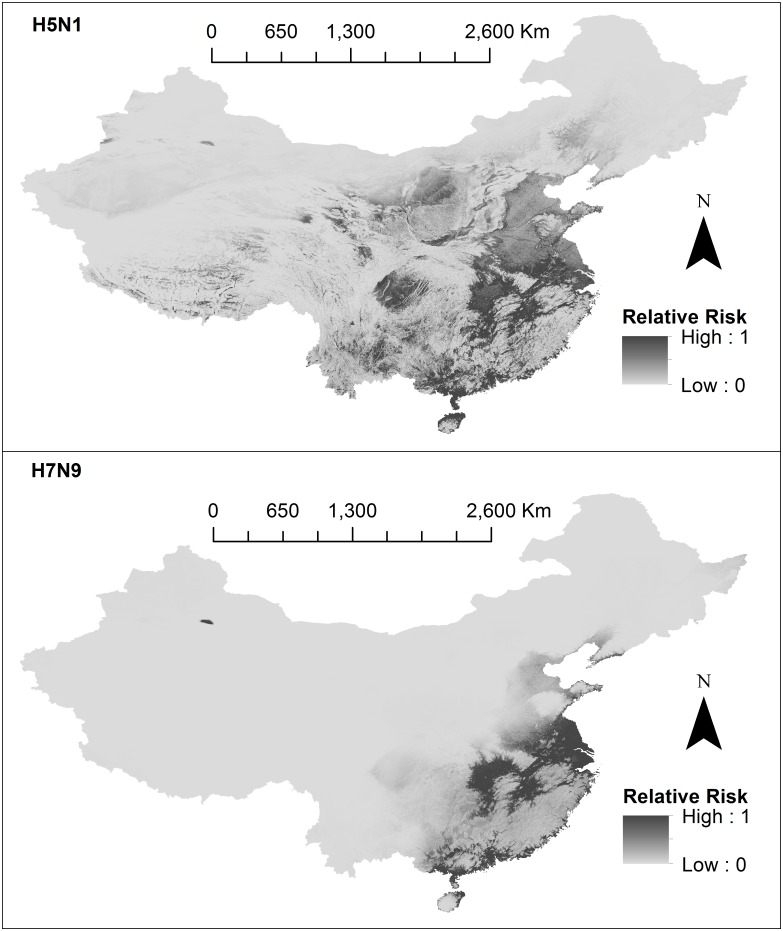
Risk models. The first panel shows H5N1 and the second panel shows H7N9. Final relative risk values for each cell (approximately 1km^2^) are represented on a continuous scale of low (light grey) to high (dark grey). Data sources used to develop risk models include: species distribution models (SDMs) 3 and 4 which were produced in this study, domestic chicken population data obtained from Livestock Geo-Wiki (http://www.livestock.geo-wiki.org/), human population data from the LandScan (2014)^™^ High Resolution global Population Data Set (http://web.ornl.gov/sci/landscan/). Base maps were obtained from the GADM database of Global Administrative Areas (http://www.gadm.org/). Maps were built using ArcMap 10.2.

Maps representing the results from this risk analysis are presented in Fig 3. For each subtype, the estimated relative risk of human infection, for each cell (approximately 1km^2^) is represented on a continuous scale from 0 (least risk) to 1 (highest risk) indicated by level of grey shading. H5N1 high risk areas were more diffusely spread around the south-eastern quarter of China, primarily in areas surrounding the Yangtze River delta. H5N1 high risk areas extended towards the Sichuan Basin, Jianghan Plain, river regions of Tibet, and Qinghai lake, as well as plains in Inner Mongolia, Shaanxi, Hebei and Henan. H7N9 high risk areas were more concentrated to the south-eastern coast line starting from Shandong and extended south towards Guangxi, encompassing nearly all of Jiangsu and Shanghai, and plains in Zhejiang, Anhui, Jiangxi, Hunan and Hubei, and Guangdong provinces.

**Fig 4:**
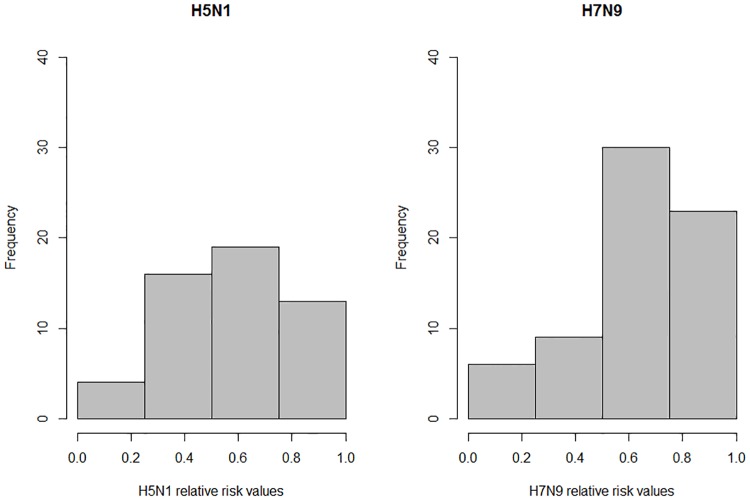
Risk model validation. Charts show the number of exact case locations which fell into a low risk area (0.0 > *R*_*k*_ ≥ 0.25), low-medium risk area (0.25 > *R*_*k*_ ≥ 0.50), medium-high risk area (0.5 > *R*_*k*_ ≥ 0.75) and high risk area (0.75 > *R*_*k*_ ≥ 1.0). The left chart shows categorisation of H5N1 exact case locations corresponding to the H5N1 risk model. The right chart shows categorisation of H7N9 exact case locations corresponding to the H7N9 risk model.

For each subtype, to validate the performance of our risk model, we extracted normalised relative risk values *R*_*k*_at exact case locations and observed where these points fell on a risk scale of low (0.0 > *R*_*k*_ ≥ 0.25), low-medium (0.25 > *R*_*k*_ ≥ 0.50), medium-high (0.5 > *R*_*k*_ ≥ 0.75) and high (0.75 > *R*_*k*_ ≥ 1.0). In Fig 4, for each subtype, we display the number of exact cases which fell into each of the four categories. The left chart shows categorisation of H5N1 exact case locations corresponding to the H5N1 risk model. The right chart shows categorisation of H7N9 exact case locations corresponding to the H7N9 risk model. For exact H5N1 cases, 7.7% (4/52) of points fell in the low risk category, 30.8% (16/52) of points fell in the low-medium risk category, 36.5% (19/52) of points fell in the medium-high risk category and 25.0% (13/52) of points fell in the high risk category. For exact H7N9 cases, 8.82% (6/68) of points fell in the low risk category, 13.2% (9/68) of points fell in the low-medium risk category, 44.1% (30/68) of points fell in the medium-high risk category and 33.8% (23/68) of points fell in the high risk category. Based on these results, the H7N9 risk model fitted more to our data set than H5N1. Human population data from LandScan (2014)^™^ for China had the smallest raster extent, hence our final risk analysis models were clipped to match this extent. One exact H7N9 case did not fall within this extent and hence was excluded from validation tests.

We additionally validated our risk model using *unexact* locations of H5N1 and H7N9 cases, however as we don't know the exact location for the unexact cases, we recorded the maximum *R*_*k*_ within a 5km radius of each unexact case (see S8 Fig). For H5N1, 20.9% (45/215) of points fell in the low risk category, 13.4% (29/215) of points fell in the low-medium risk category, 37.2% (80/215) of points fell in the medium-high risk category and 28.3% (61/215) of points fell in the high risk category. For H7N9 15.3% (187/1221) of points fell in the low risk category, 21.5% (262/1221) of points fell in the low-medium risk category, 39.7% (485/1221) of points fell in the medium-high risk category and 23.5% (287/1221) of points fell in the high risk category.

**Fig 5:**
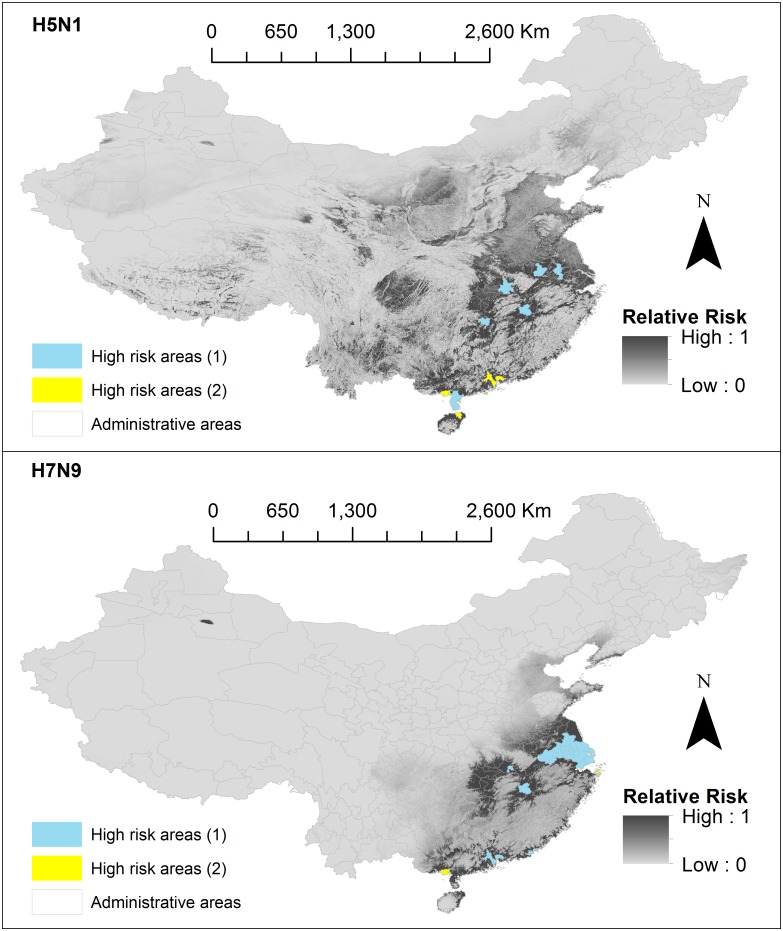
High risk areas. The first panel shows H5N1 and the second panel shows H7N9. Final relative risk values for each cell (approximately 1km^2^) are represented on a continuous scale of low (light grey) to high (dark grey). High risk areas (1) represent secondary administrative areas (prefectures, municipalities, cities) areas with a mean-aggregated relative risk value (> 0.5), and high risk areas (2) are those areas which have not yet reported a case. Data sources used to develop risk models include: species distribution models (SDMs) 3 and 4 which were produced in this study, domestic chicken population data obtained from Livestock Geo-Wiki (http://www.livestock.geo-wiki.org/), human population data from the LandScan (2014)^™^ High Resolution global Population Data Set (http://web.ornl.gov/sci/landscan/). Base maps of Chinese administrative regions were obtained from the GADM database of Global Administrative Areas (http://www.gadm.org/). Maps were built using ArcMap 10.2.

Final relative risk values, *R*_*k’*_were aggregated across all secondary administrative areas (prefectures, municipalities, cities) to quantify the relative risk posed to a particular area. The mean value per area was taken as the aggregated relative risk value. High risk areas were considered as those with a mean > 0.5 and are highlighted in Fig 5. For H5N1, these areas included: Foshan, Zhongshan, Dongguan, Zhanjiang (Guangdong); Beihai (Guangxi); Ezhou, Wuhan (Hubei); Nanchang (Jiangxi); Nanjing (Jiangsu); Hefei (Anhui); Xiangtan (Hunan); and Haikou (Hainan). For H7N9, these areas included: Shanghai municipality, Jiaxing, Zhoushan (Zhejiang); Foshan, Zhongshan, Shantou, Dongguan (Guangdong); Changzhou, Nantong, Suzhou, Wuxi, Taizhou, Zenjiang, Yangzhou, Nanjing (Jiangsu), Ma’anshan, Wuhu, Tongling, Chaohu (Anhui); Beihai (Guangxi); Ezhou (Hubei); and Nanchang (Jiangxi).

We also highlighted high risk areas which have not yet reported a H5N1 or H7N9 case (High risk areas (1) in Fig 5). An area is considered to have a H5N1 or H7N9 case if an exact or unexact case fell within the area. For H5N1 these included: Dongguang, Foshan and Zhongshan (Guangdong); Beihai (Guangxi); and Haikou (Hainan). For H7N9 these included: Beihai (Guangxi) and Zhoushan (Zhejiang). The computed risk map can also be downloaded from S3 and S4 Files in ASCII format.

In previous SDMs, LBM density showed strong associations with H7N9 [27,28,30] and H5N1 [30]. LBMs are locations where AIV transmission can be especially amplified due to the mixing of chickens from different farms, overcrowding, and stressful (and thus immunosuppressive) poultry housing conditions. Inclusion of this variable into the risk analysis would account for the high transmission intensity seen in LBM environments.

**Fig 6:**
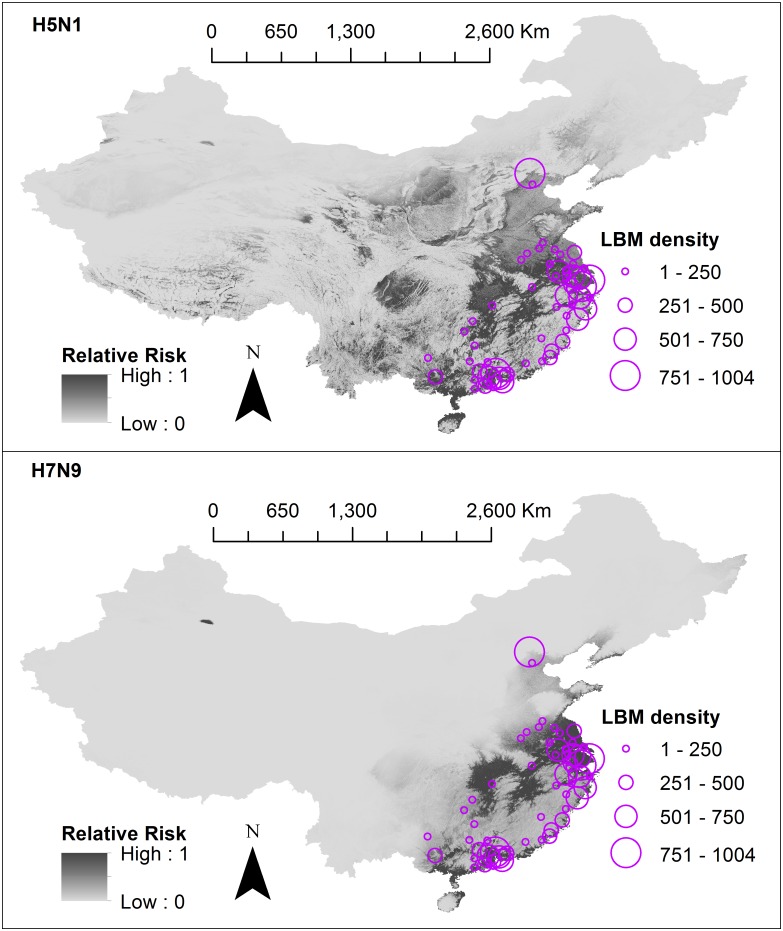
Risk models overlayed with live bird market density. The first panel shows H5N1 and the second panel shows H7N9. Final relative risk values for each cell (approximately 1km^2^) are represented on a continuous scale of low (light grey) to high (dark grey). Different sized circles represent the live bird market density per secondary administrative areas (prefectures, municipalities, cities), however data were only available for 43 (of 344) of these areas. Data sources used to develop risk models include: species distribution models (SDMs) 3 and 4 which were produced in this study, domestic chicken population data obtained from Livestock Geo-Wiki (http://www.livestock.geo-wiki.org/), human population data from the LandScan (2014)^™^ High Resolution global Population Data Set (http://web.ornl.gov/sci/landscan/). Live bird market data were requested from authors of previously published SDMs [27,30]. Base maps of Chinese administrative regions were obtained from the GADM database of Global Administrative Areas (http://www.gadm.org/). Maps were built using ArcMap 10.2.

We were able to collect LBM data (from [27,30], previously purchased from a commercial mapping provider www.autonavi.com), however data collection methods were not available for scrutiny. Due to uncertainties regarding reliability and validity of this data set, we did not incorporate a LBM variable into the formal risk model. Instead, we presented our risk models overlayed with LBM data aggregated to secondary administrative areas (see Fig 6). LBM data was only available for 43 (of 344) prefectures, municipalities and cities within 10 (of 31) provinces in China. Only 10 (of 52) H5N1 exact points and 45 (of 69) H7N9 exact points fell into these regions. Secondary administrative areas with highest LBM counts included Jiaxing (Zhejiang), Beijing, Guangzhou (Guangdong) and Shanghai.

## Discussion

In this study, we (i) developed a refined data set of H5N1 and H7N9 locations with consideration of animal/animal environment case data as well as spatial accuracy and precision of coordinates; (ii) used this data set along with environmental variables in Maxent to build SDMs for each subtype in high resolution spatial units of 1km^2^ cells; (iii) developed a risk analysis framework, which integrated Maxent models with human and chicken population density to estimate the geographic risk of zoonotic transmission of H5N1 and H7N9; and (iv) identified areas of China at high risk of H5N1 and H7N9 transmission differentiated into two groups, those that have and have not yet reported cases.

From our risk models, areas at high risk of both H5N1 and H7N9 transmission were identified along the south-eastern coast starting from Shandong province and extended south towards Guangxi province. These areas include traditional rice-duck farming areas of Huzhou and Hangzhou city (Zhejiang), and communities around Taihu lake and Qiantang river [28], traditional rice-duck farming systems create an ideal environment for AIV dissemination because of the mixing of semi-domestic and wild duck hosts which are the traditional carriers of AIVs [8]. Duck farming systems exist widely in villages in coastal southern areas [37,69] and are known high risk areas for AIV transmission [28,70,71]. Inland, both H5N1 and H7N9 risk models demonstrate high risk in areas surrounding the Yangtze River delta, including its major tributaries such as Dongting Lake and Poyang Lake. These are migratory bird resting sites, and are also known high risk areas for AIV transmission [8]. Our risk models appropriately do not estimate high risk in large expanses of uninhabitable regions that cannot support maintenance of virus (e.g. expansive deserts in Inner Mongolia, Tibet and Xinjiang autonomous regions as identified in Fang et al. [71]).

The final H5N1 and H7N9 risk models differed in terms of their respective geographic profiles, and in terms of performance. For both subtypes, high risk areas of transmission were identified in mainly south-eastern China, with H5N1 distribution extending more inland into Jiangxi province. These results were consistent with Li et al. [72] (the only other study to build H5N1 and H7N9 models using comparable means). Both H5N1 and H7N9 risk models showed reasonable predictive capacity, with the majority of exact points falling into high and medium-high risk areas (61.5% for H5N1 and 77.9% for H7N9). However fewer points fell into high risk categories (25% for H5N1 and 33.8% for H7N9). The predictive capacity for the H5N1 risk model was lower for the H7N9 model, likely due to the influence of the *p*_*k*_ value (i.e. the suitability value).

Despite potential sampling biases, and a relatively small number of case data, we produced high performing SDMs (6 of 8 models with AUC > 0.9) which are similar to previous H5N1 [31,37] and H7N9 SDMs [24–30]. In all our SDMs, H7N9 consistently showed higher AUC results compared to H5N1, suggesting H7N9 suitability models could be better explained by seasonality and land coverage. This could be associated with the differences in H7N9 and H5N1 temporal distributions: H7N9 exhibits high seasonality (cases consistent peaking around January to February), whereas H5N1 exhibits a more sporadic distribution pattern [3].

The seasonal pattern of H7N9 is thought to be correlated to increases in poultry stock and sales during Chinese New Year festivities [73] however, this would not be implicit to only one AIV subtype. Alternatively, there is also a likely difference in the completeness of our H5N1 and H7N9 case datasets. For H5N1, most exact records were drawn from unknown location types (17 of 52) whereas for H7N9, most (54 of 69) exact H7N9 cases were obtained from LBMs where most H7N9 zoonotic transmission takes place. Our exact H7N9 cases were mostly concentrated in Guangdong (20 of 29) and Zhejiang (11 of 69) whereas H5N1 cases are more evenly spread across the 17 primary administrative areas. Our H5N1 exact records were evenly distributed temporally with most cases (30 of 52) being drawn from 2004 and 2014, however we would expect to find more H5N1 cases in the years before widescale vaccination campaigns in poultry from 2004 to 2008. The time lag between the emergence of H5N1 and H7N9 means that H7N9 case reporting and identification is likely to be more complete, due to advances in AIV control and surveillance capabilities and technologies over the years.

There is likely increased transmission intensity within LBMs, hence LBM density would be an important parameter to include in our risk model. However, we were unable to ascertain how collection of LBM locations was performed and whether sampling bias contributed significantly to higher LBM densities in Jiaxing (Zhejiang), Beijing, Guangzhou (Guangdong) and Shanghai. From a simple visual appraisal, areas of high risk of H5N1 and H7N9 fell into areas where there are high numbers of LBM with the exception of Beijing. Isolations of H5N1 and H7N9 have been infrequent in Beijing, it is likely that the high density of LBMs found in this region is possibly due to data collection being more easily performed in the major cities (however we were unable to ascertain how the data collection was performed).

Our risk model aimed to identify specific geographic areas of high risk—however there are other modelling methodologies which explicitly model disease spread within populations. A commonly used example is the dynamical epidemic SIR model, which uses ordinary differential equations to forecast how host populations transition through biologically relevant disease states (e.g. Susceptible, Infectious, Recovered) over a time period [65]. In contrast to our study, these models are able to provide an indication of whether the disease is capable of sustaining itself in a human population (indicated when a computed basic reproduction number (R_0_) >1), and also evaluate effectiveness of public health interventions. However many of these studies don’t distinguish the differing risks inherent in different geographic areas at particularly high resolutions [74–78]. Typically, only spatial resolutions at the country or province level are used.

In Li et al. [77], the authors aggregated their model into two spatial regions which encapsulated multiple provinces e.g. the southern region included Guangdong, Fujian and Hunan provinces, and the eastern region included Jiangsu, Zhejiang, Shanghai and Anhui provinces. They estimated the transmission potential of H7N9 among chicken flocks and found differences between the two regions—in the eastern region, initial chicken host susceptibility decreased over time, but in the southern region susceptibility remained stable. In our study, we showed that most medium-high and high risk regions for H7N9 were primarily in eastern provinces (6/24), compared to the southern provinces (16/24), with a few medium-high and high risk regions identified inland (n = 2). We found relative risk values were overall higher in eastern provinces (mean *R*_*k*_ of Jiangsu, Zhejiang, Shanghai and Anhui provinces ranged from 0.24 to 0.72) compared to southern provinces (mean *R*_*k*_ of Guangdong, Fujian and Hunan provinces ranged from 0.11 to 0.23). Future works could attempt to integrate both dynamical models with spatial risk models to take into account both disease dynamics and inherent geographic heterogeneity.

Novel methods which use the SIR framework to quantify the spatial pattern transitions of disease spread are currently being studied (described in detail in [79,80]). For example, Li [79] describes the phenomenon of patch invasion, which can be determined using susceptible-infectious host dynamics, and like the R_0_, can indicate whether a disease will maintain itself in isolated geographic distributions as opposed to spreading into other geographic regions. Further development of such methodologies would be useful to consider in future spatial models of epidemics.

This study is subject to certain limitations, which are discussed in detail below.

1. A bias for AIV reporting may occur because areas of high human density often have more resources to implement active and passive surveillance of AIV. Thus, our models may have overestimated the risk posed to areas with high human density. This may also partially explain why our SDMs and risk models, which include a human population variable, are highly similar. Likewise, there is a known paucity of AIV surveillance in more uninhabitable areas of northern and western China—hence our SDMs may have underestimated risk in these areas. Herrick et al. [59] solely identify the entire north-eastern corner to have high AIV suitability, albeit their study focused only on AIV in wild bird hosts.
2. The range of possible virus hosts for AIVs is uncertain; *e*.*g*., H5N1 has occasionally been identified in wild ducks, geese and swans, while H7N9 has not yet been identified in wild birds. In this study we did not conduct separate analyses by host type due to limitations in available data, *i*.*e*., this would result in too few cases (see S1 and S2 Tables for breakdowns of animal host). Therefore, our study did not take into consideration the differences in habitat suitability for the wide range of AIV host species (e.g. domestic poultry, domestic ducks, wild ducks and geese, humans and other potential carriers of AIV), nor the differences in virus shedding and transmission in different host species [5].
3. There are limitations inherent in resampling and manipulation of the chicken and human density data sources. As described in Feng et al. [81], log transformation of original data precludes our ability to make inferences regarding the original data.
4. Species distribution models inherently only predict probable presence or absence, and do not predict species abundance. Abundance of H5N1 and H7N9 in avian populations is considered to be extremely low as determined in a previous meta-analysis of H5N1 and H7N9 surveillance studies [6]. In incidental hosts (i.e. human populations) and the environment, prevalence is expected to be much lower. As such, our risk models may have over-estimated actual risk.
5. Our analysis only accounted for the risk of autochthonous AIV transmission. Risk of infection can also occur through human travel and poultry trading.
6. Limitations inherent in this risk assessment methods are similar to those described for MCDA [32,82], which include (i) overweighing variables from correlations between different environmental variables, and correlations between environmental and population variables, and (ii) subjectivity in the selection of variables, formulation of risk equation and selection of fuzzy membership functions and parameters.
7. Our risk analysis implied that domestic chickens are the sole animal population responsible for AIV transmission even though both H5N1 and H7N9 have been recognised in various other avian and mammalian species. Our rationale for focusing on chickens is described in the methods section above. We chose to exclude duck density because in some China-only models, duck density had either not been used as a distinct variable [27,30], or when used, was not deemed an important contributor [28]. While it is likely that domestic duck density also contributes to the spread of H5N1, limited disease occurrence data (outbreak data rather than active surveillance data) makes it difficult to accurately model its impact [37].
8. This analysis did not consider certain transmission dynamics which are important in the disease cycle. Transmission efficiency is modulated by the degree and strength of contact between poultry and humans, as well as the inherent (i.e. genetic) efficiency of the virus to attach and replicate in humans. The level of animal handling, personal protection (e.g. masks, gloves), or biosecurity measures are currently not collected on a large scale; thus accounting for such transmission dynamics was beyond the scope of this paper. In addition, while there are certain genetic mutations known to promote attachment and replication to human cells, computationally incorporating these genetic influences into our risk model was also beyond the scope of this study.
9. Our study did not take into account the wide range of competing AIV subtypes, nor did it differentiate between strains within a subtype.

## Conclusion

H5N1 and H7N9 subtypes have persisted in animal hosts and continue to cause human infections since their emergence in China. Additionally, novel zoonotic AIVs (such as H10N8 and H5N6) have also emerged in mainland China, and human infections from these subtypes have so far been restricted to China. Furthermore, surveillance of poultry reveals there are many new reassortant AIVs being discovered [83,84]. Hence, zoonotic avian influenza poses a major risk to China.

As outlined previously in the introduction, the motivation for this study was to identify specific areas to target and trial control measures which cannot be implemented on a large scale due to cost or public disapproval. Quantifiable reductions of H7N9 human cases and AIV environmental samples have already been demonstrated following closure of LBMs in cities in Guangzhou (Guangdong); Shanghai; Hangzhou, Huzhou (Zhejiang); and Nanjing (Jiangsu) [85–90]. Peiris et al. [91] provide an extensive discussion of practical measures that can be made (temporary LBM rest days, removing live poultry from holdings overnight, separating aquatic poultry from gallinaceous poultry, centralised slaughtering of poultry), we recommend implementing these control measures in the high risk secondary administrative areas identified in our H5N1 and H7N9 risk models: Foshan, Zhongshan, Shantou, Dongguan, Zhanjiang (**Guangdong**); Beihai (**Guangxi**); Wuhan, Ezhou (**Hubei**); Nanchang (**Jiangxi**); Ma’anshan, Wuhu, Tongling, Chaohu, Hefei (**Anhui**); Xiangtan (**Hunan**); Jiaxing, Zhoushan (**Zhejiang**); Changzhou, Nantong, Suzhou, Wuxi, Taizhou, Zenjiang, Yangzhou (**Jiangsu**); and Haikou (**Hainan**). The results from the risk models provide high resolution risk projections, which allow appreciable discrimination of higher risk areas within these regions (available for download: S3 and S4 Files) to further target implementation of control measures.

SDMs are advantageous over simple mapping techniques, as they provide a spatially continuous estimate of disease presence, and are able to identify areas at risk of transmission where cases have not previously been recognised. A reliable risk model can reduce the need for expensive, large-scale surveillance programs to identify high risk areas. Our study finds differences in the geographic distribution and performance of H5N1 and H7N9 SDMs suggesting there may be intrinsic differences in how the novel H7N9 survives in the environment. We suggest future research focus on comparing H5N1 and H7N9 prevalence rates and survivability in the natural environment to develop a better understanding of environmental transmission in human infection.

## Supporting information

S1 Fig Exact H5N1 cases: spatial distribution in China. First panel indicates distribution by year, middle panel indicates distribution by type of host, last panel indicates type of location. Chinese provinces are outlined in grey. Data sources used to obtain the case locations include: the Food and Agricultural Organization (FAO) (http://empres-i.fao.org/eipws3g/), the Chinese Ministry of Agriculture Avian Influenza Surveillance Reports (www.syj.moa.gov.cn), the World Organization of Animal Health (OIE) reports (www.oie.int). Base maps were obtained from the GADM database of Global Administrative Areas (http://www.gadm.org/). Maps were built using ArcMap 10.2.
(TIF)

S2 Fig Exact H7N9 cases: spatial distribution in China. First panel indicates distribution by year, middle panel indicates distribution by type of host, last panel indicates type of location. Chinese provinces are outlined in grey. Data sources used to obtain the case locations include: the Food and Agricultural Organization (FAO) (http://empres-i.fao.org/eipws3g/), the Chinese Ministry of Agriculture Avian Influenza Surveillance Reports (www.syj.moa.gov.cn), the World Organization of Animal Health (OIE) reports (www.oie.int). Base maps were obtained from the GADM database of Global Administrative Areas (http://www.gadm.org/). Maps were built using ArcMap 10.2.
(TIF)

S3 Fig Sub-selection of provinces for species distribution models 5–8. Map showing the 22 (of 31) primary administrative regions (provinces, municipalities, autonomous regions) selected as the study area in constructing SDM 5–8 (in grey). Base maps were obtained from the GADM database of Global Administrative Areas (http://www.gadm.org/). Maps were built using ArcMap 10.2.
(TIF)

S4 Fig Risk analysis variables. Top row *c*_*k*_values at cells enclosing H5N1 and H7N9 exact points; bottom row *h*_*k*_values at cells enclosing H5N1 and H7N9 exact points.
(TIF)

S5 Fig Species distribution models 1–2. The first panel shows H5N1 (SDM 1) and the second panel shows H7N9 (SDM 2). Suitability values for each cell (approximately 1km^2^) are represented on a continuous scale of low (light grey) to high (dark grey). SDMs were built using Maxent software version 3.3.3k (available from https://www.cs.princeton.edu/~schapire/maxent/). SDMs were developed using environmental variables, created using data from: the WorldClim database (www.wordlclim.org), the Shuttle Radar Topography Mission (SRTM) 90m Digital Elevation Database v4.1 (www.cgiar-csi.org). Data sources used to obtain the case locations to build SDMs include: the Food and Agricultural Organization (FAO) (http://empres-i.fao.org/eipws3g/), the Chinese Ministry of Agriculture Avian Influenza Surveillance Reports (www.syj.moa.gov.cn), the World Organization of Animal Health (OIE) reports (www.oie.int). Base maps were obtained from the GADM database of Global Administrative Areas (http://www.gadm.org/). Maps were built using ArcMap 10.2.
(TIF)

S6 Fig Species distribution models 5–6. The first panel shows H5N1 (SDM 5) and the second panel shows H7N9 (SDM 6). Suitability values for each cell (approximately 1km^2^) are represented on a continuous scale of low (light grey) to high (dark grey). SDMs were built using Maxent software version 3.3.3k (available from https://www.cs.princeton.edu/~schapire/maxent/). SDMs were developed using environmental variables, created using data from: the WorldClim database (www.wordlclim.org), the Shuttle Radar Topography Mission (SRTM) 90m Digital Elevation Database v4.1 (www.cgiar-csi.org). Data sources used to obtain the case locations to build SDMs include: the Food and Agricultural Organization (FAO) (http://empres-i.fao.org/eipws3g/), the Chinese Ministry of Agriculture Avian Influenza Surveillance Reports (www.syj.moa.gov.cn), the World Organization of Animal Health (OIE) reports (www.oie.int). Base maps were obtained from the GADM database of Global Administrative Areas (http://www.gadm.org/). Maps were built using ArcMap 10.2.
(TIF)

S7 Fig Species distribution models 7–8. The first panel shows H5N1 (SDM 7) and the second panel shows H7N9 (SDM 8). Suitability values for each cell (approximately 1km^2^) are represented on a continuous scale of low (light grey) to high (dark grey). SDMs were built using Maxent software version 3.3.3k (available from https://www.cs.princeton.edu/~schapire/maxent/). SDMs were developed using environmental variables, created using data from: the WorldClim database (www.wordlclim.org), the Shuttle Radar Topography Mission (SRTM) 90m Digital Elevation Database v4.1 (www.cgiar-csi.org). Data sources used to obtain the case locations to build SDMs include: the Food and Agricultural Organization (FAO) (http://empres-i.fao.org/eipws3g/), the Chinese Ministry of Agriculture Avian Influenza Surveillance Reports (www.syj.moa.gov.cn), the World Organization of Animal Health (OIE) reports (www.oie.int). Base maps were obtained from the GADM database of Global Administrative Areas (http://www.gadm.org/). Maps were built using ArcMap 10.2.
(TIF)

S8 Fig Risk model validation for all cases using aggregated relative risk values. Number of all (unexact and exact) points per risk category (low 0.0–0.25; low-medium 0.25–0.50; medium-high 0.50–0.75; high 0.75–1.00). For each point, the maximum 1km cell relative risk value within approximately 5km radius was taken as the aggregated relative risk value.
(TIF)

S1 Table Summary of H5N1 exact locations. (DOCX)

S2 Table Summary of H7N9 exact locations. (DOCX)

S3 Table Summary of variables used in risk analysis. (DOCX)

S1 File Data set of exact H5N1 case locations. Each entry represents a case location which we considered an ‘exact’ case location. For each entry, we provide the source of information, and information associated with the case e.g. latitude and longitude coordinates, date associated with poultry outbreak or human case, the host species associated with the sample.
(CSV)

S2 File Data set of exact H7N9 case locations. Each entry represents a case location which we considered an ‘exact’ case location. For each entry, we provide the source of information, and information associated with the case e.g. latitude and longitude coordinates, date associated with poultry outbreak or human case, the host species associated with the sample.
(CSV)

S3 File H5N1 risk assessment map. Final computed H5N1 relative risk values for each cell (approximately 1km^2^) in China in ASCII format.
(ZIP)

S4 File H7N9 risk assessment map. Final computed H7N9 relative risk values for each cell (approximately 1km^2^) in China in ASCII format.
(ZIP)
