# Correction: Influenza A H5N1 and H7N9 in China: A spatial risk analysis

**DOI:** 10.1371/journal.pone.0176903

**Published:** 2017-04-27

**Authors:** Chau Minh Bui, Lauren Gardner, C. Raina MacIntyre, Sahotra Sarkar

The third author’s name is incorrect. The correct name is: C. Raina MacIntyre. The correct citation is: Bui CM, Gardner L, MacIntyre CR, Sarkar S (2017) Influenza A H5N1 and H7N9 in China: A spatial risk analysis. PLoS ONE 12(4): e0174980. https://doi.org/10.1371/journal.pone.0174980
