# Supplementary figures and images for: Influenza A H5N1 and H7N9 in China: A spatial risk analysis
Source: PLoS One. 2017 Apr 4;12(4):e0174980. doi: 10.1371/journal.pone.0174980 (PMC5380336; doi:10.1371/journal.pone.0174980)

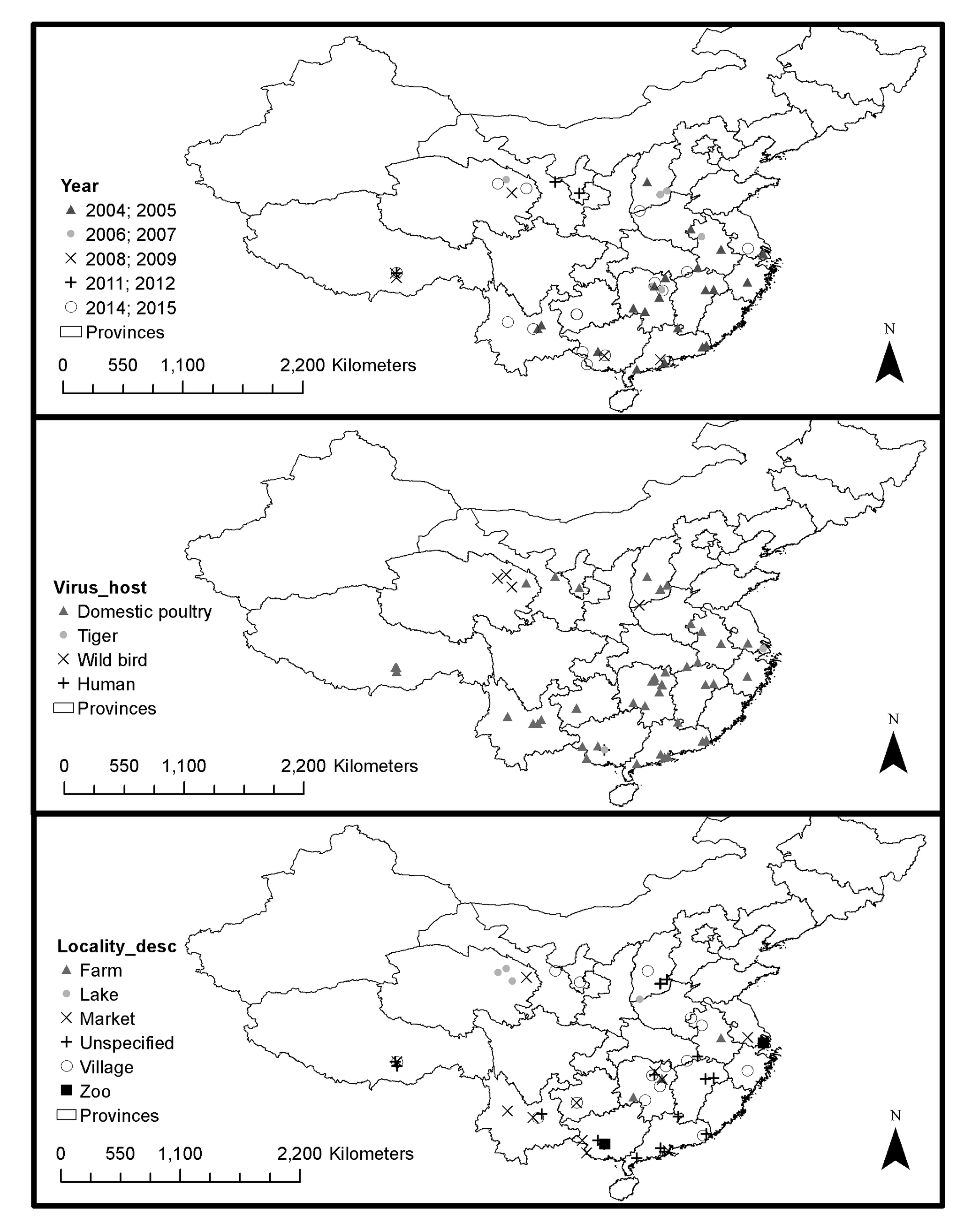

Supplement: S1 Fig — First panel indicates distribution by year, middle panel indicates distribution by type of host, last panel indicates type of location. Chinese provinces are outlined in grey. Data sources used to obtain the case locations include: the Food and Agricultural Organization (FAO) (http://empres-i.fao.org/eipws3g/), the Chinese Ministry of Agriculture Avian Influenza Surveillance Reports (www.syj.moa.gov.cn), the World Organization of Animal Health (OIE) reports (www.oie.int). Base maps were obtained from the GADM database of Global Administrative Areas (http://www.gadm.org/). Maps were built using ArcMap 10.2. (TIF) [file pone.0174980.s001.tif]

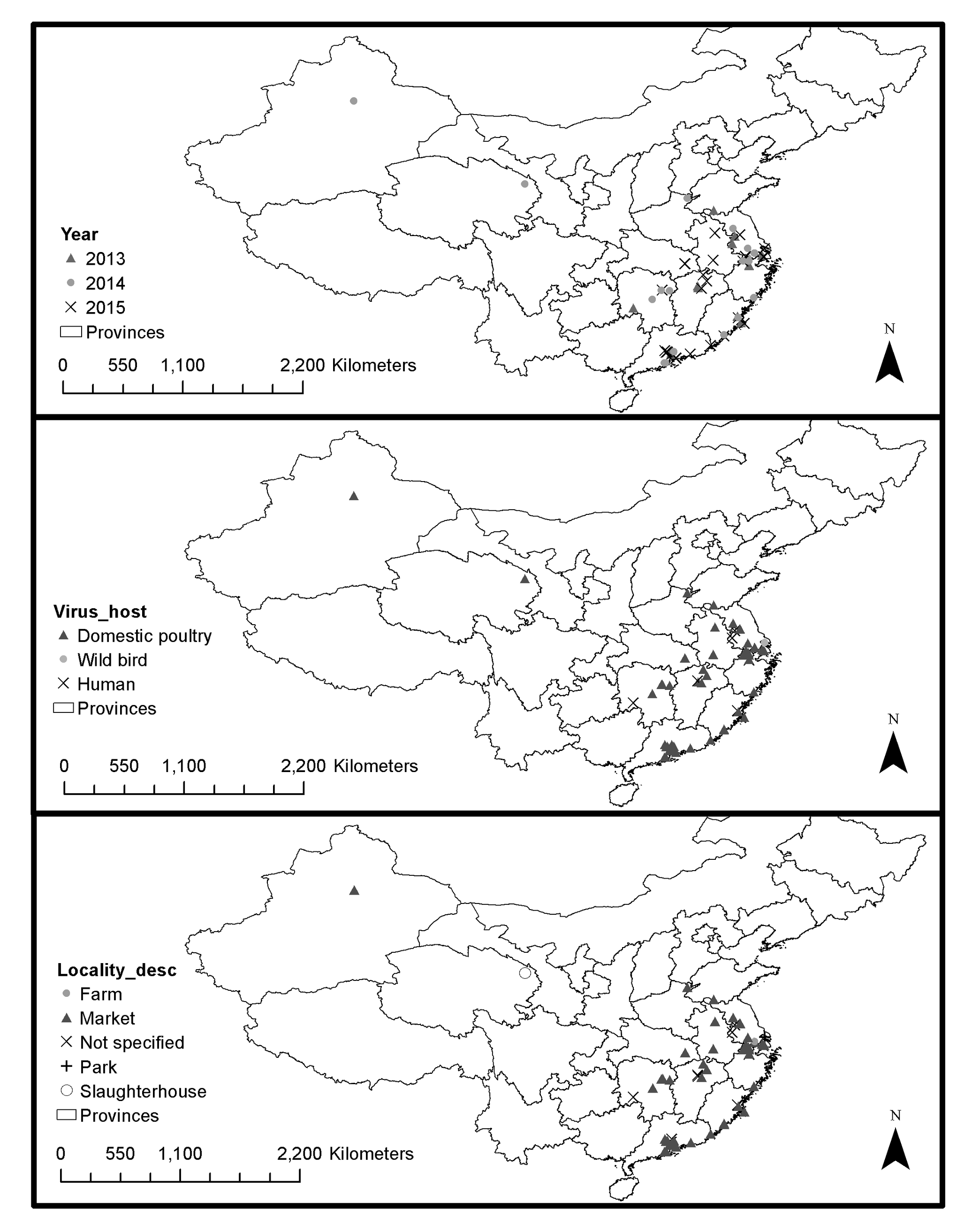

Supplement: S2 Fig — First panel indicates distribution by year, middle panel indicates distribution by type of host, last panel indicates type of location. Chinese provinces are outlined in grey. Data sources used to obtain the case locations include: the Food and Agricultural Organization (FAO) (http://empres-i.fao.org/eipws3g/), the Chinese Ministry of Agriculture Avian Influenza Surveillance Reports (www.syj.moa.gov.cn), the World Organization of Animal Health (OIE) reports (www.oie.int). Base maps were obtained from the GADM database of Global Administrative Areas (http://www.gadm.org/). Maps were built using ArcMap 10.2. (TIF) [file pone.0174980.s002.tif]

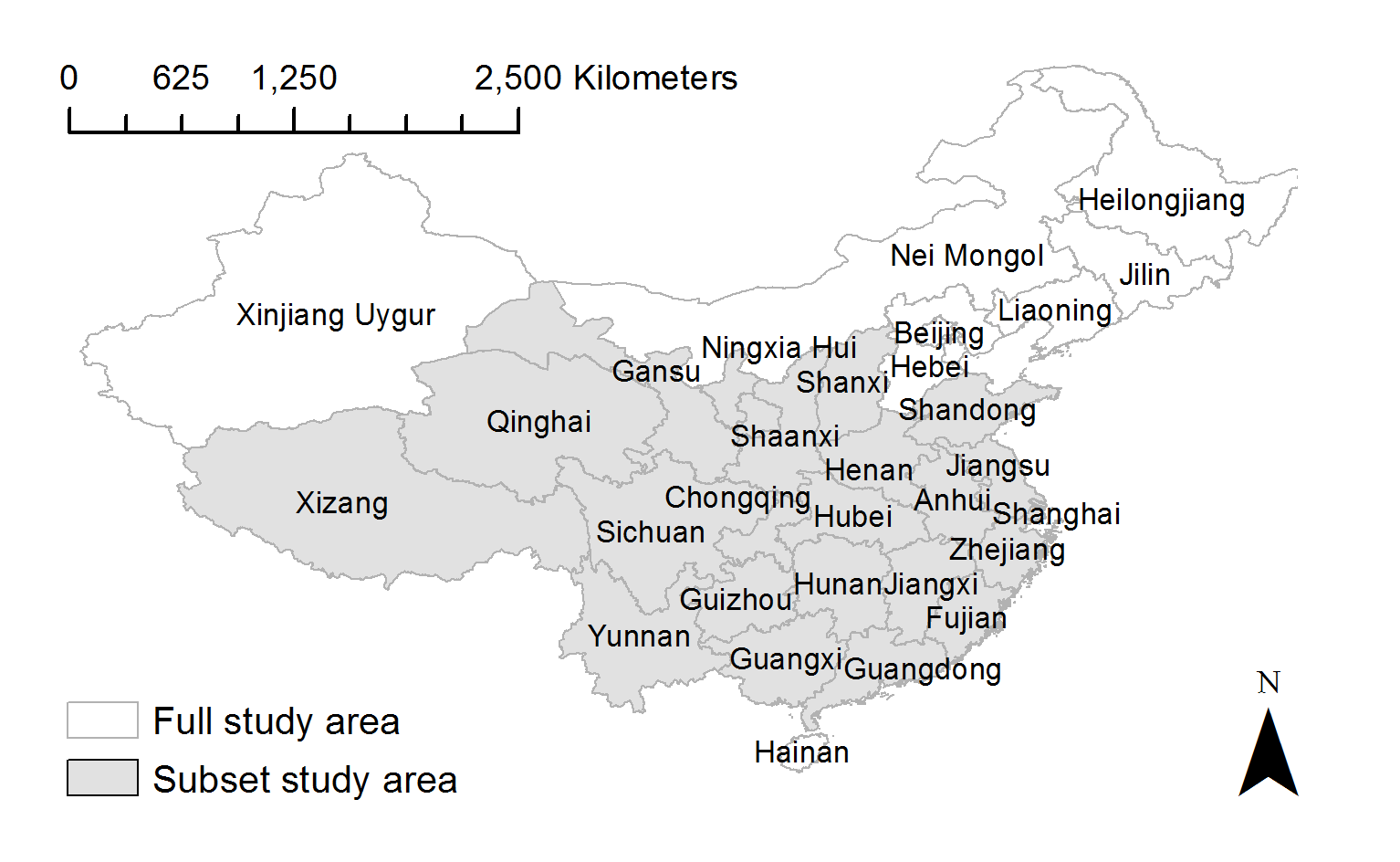

Supplement: S3 Fig — Map showing the 22 (of 31) primary administrative regions (provinces, municipalities, autonomous regions) selected as the study area in constructing SDM 5–8 (in grey). Base maps were obtained from the GADM database of Global Administrative Areas (http://www.gadm.org/). Maps were built using ArcMap 10.2. (TIF) [file pone.0174980.s003.tif]

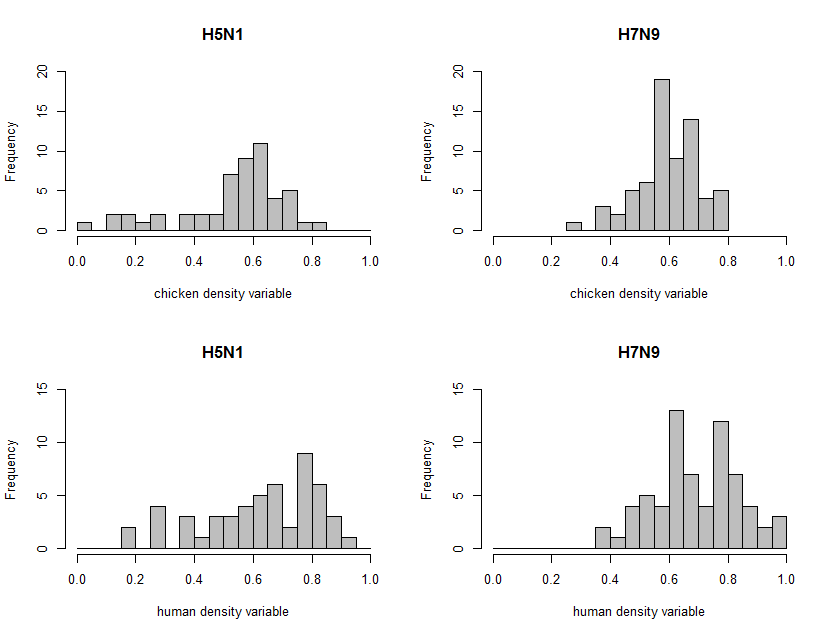

Supplement: S4 Fig — Top row ckvalues at cells enclosing H5N1 and H7N9 exact points; bottom row hkvalues at cells enclosing H5N1 and H7N9 exact points. (TIF) [file pone.0174980.s004.tif]

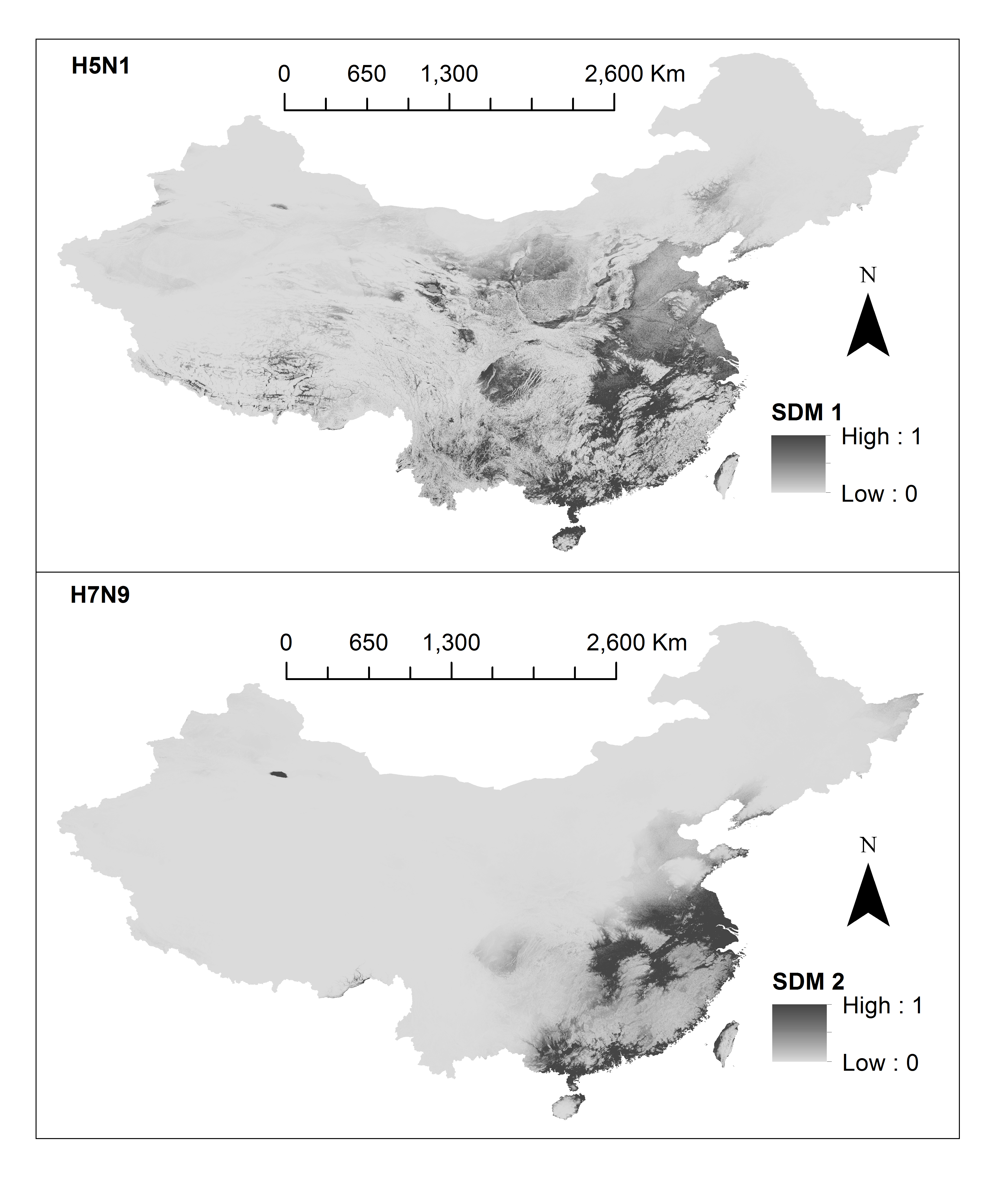

Supplement: S5 Fig — The first panel shows H5N1 (SDM 1) and the second panel shows H7N9 (SDM 2). Suitability values for each cell (approximately 1km2) are represented on a continuous scale of low (light grey) to high (dark grey). SDMs were built using Maxent software version 3.3.3k (available from https://www.cs.princeton.edu/~schapire/maxent/). SDMs were developed using environmental variables, created using data from: the WorldClim database (www.wordlclim.org), the Shuttle Radar Topography Mission (SRTM) 90m Digital Elevation Database v4.1 (www.cgiar-csi.org). Data sources used to obtain the case locations to build SDMs include: the Food and Agricultural Organization (FAO) (http://empres-i.fao.org/eipws3g/), the Chinese Ministry of Agriculture Avian Influenza Surveillance Reports (www.syj.moa.gov.cn), the World Organization of Animal Health (OIE) reports (www.oie.int). Base maps were obtained from the GADM database of Global Administrative Areas (http://www.gadm.org/). Maps were built using ArcMap 10.2. (TIF) [file pone.0174980.s005.tif]

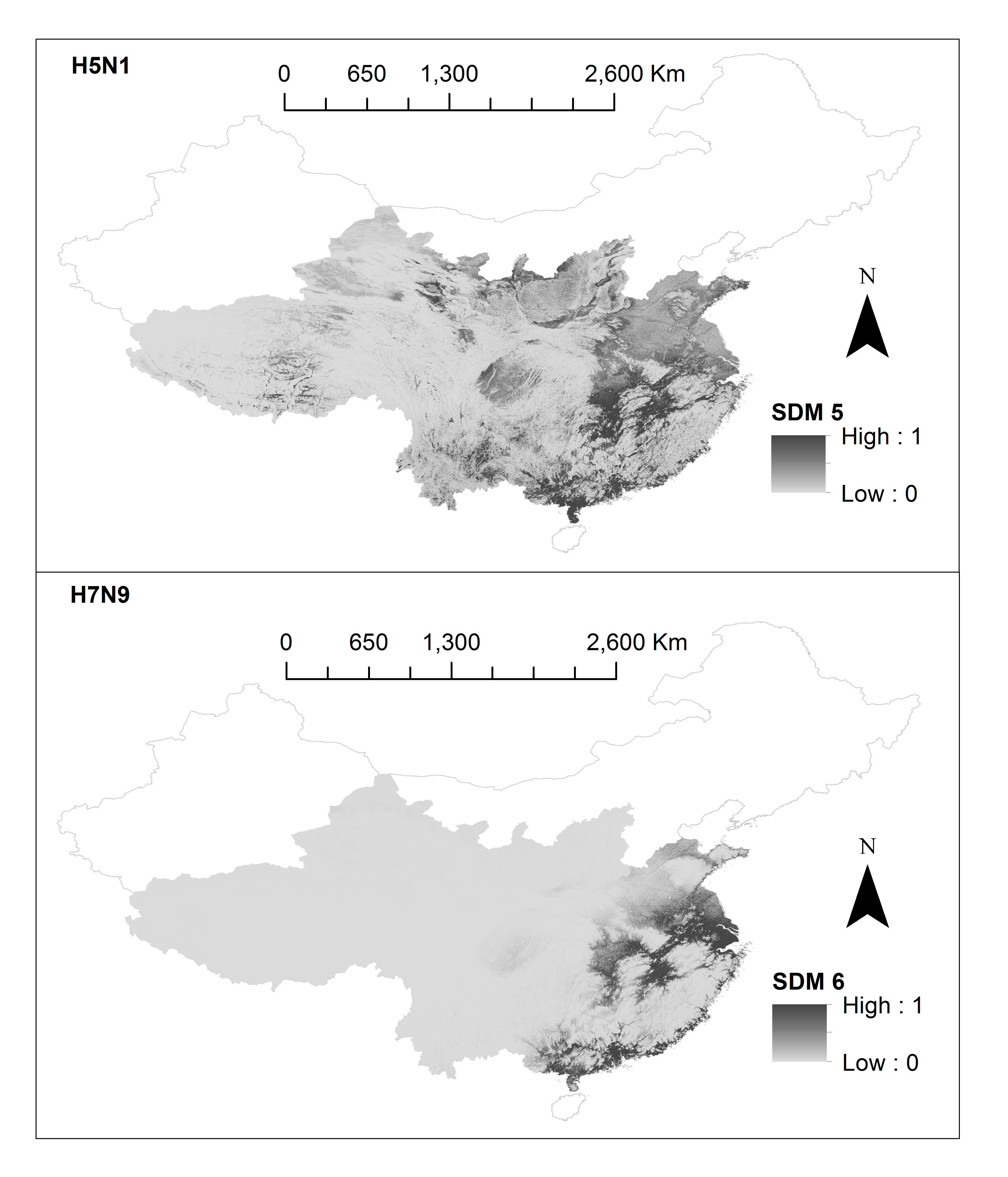

Supplement: S6 Fig — The first panel shows H5N1 (SDM 5) and the second panel shows H7N9 (SDM 6). Suitability values for each cell (approximately 1km2) are represented on a continuous scale of low (light grey) to high (dark grey). SDMs were built using Maxent software version 3.3.3k (available from https://www.cs.princeton.edu/~schapire/maxent/). SDMs were developed using environmental variables, created using data from: the WorldClim database (www.wordlclim.org), the Shuttle Radar Topography Mission (SRTM) 90m Digital Elevation Database v4.1 (www.cgiar-csi.org). Data sources used to obtain the case locations to build SDMs include: the Food and Agricultural Organization (FAO) (http://empres-i.fao.org/eipws3g/), the Chinese Ministry of Agriculture Avian Influenza Surveillance Reports (www.syj.moa.gov.cn), the World Organization of Animal Health (OIE) reports (www.oie.int). Base maps were obtained from the GADM database of Global Administrative Areas (http://www.gadm.org/). Maps were built using ArcMap 10.2. (TIF) [file pone.0174980.s006.tif]

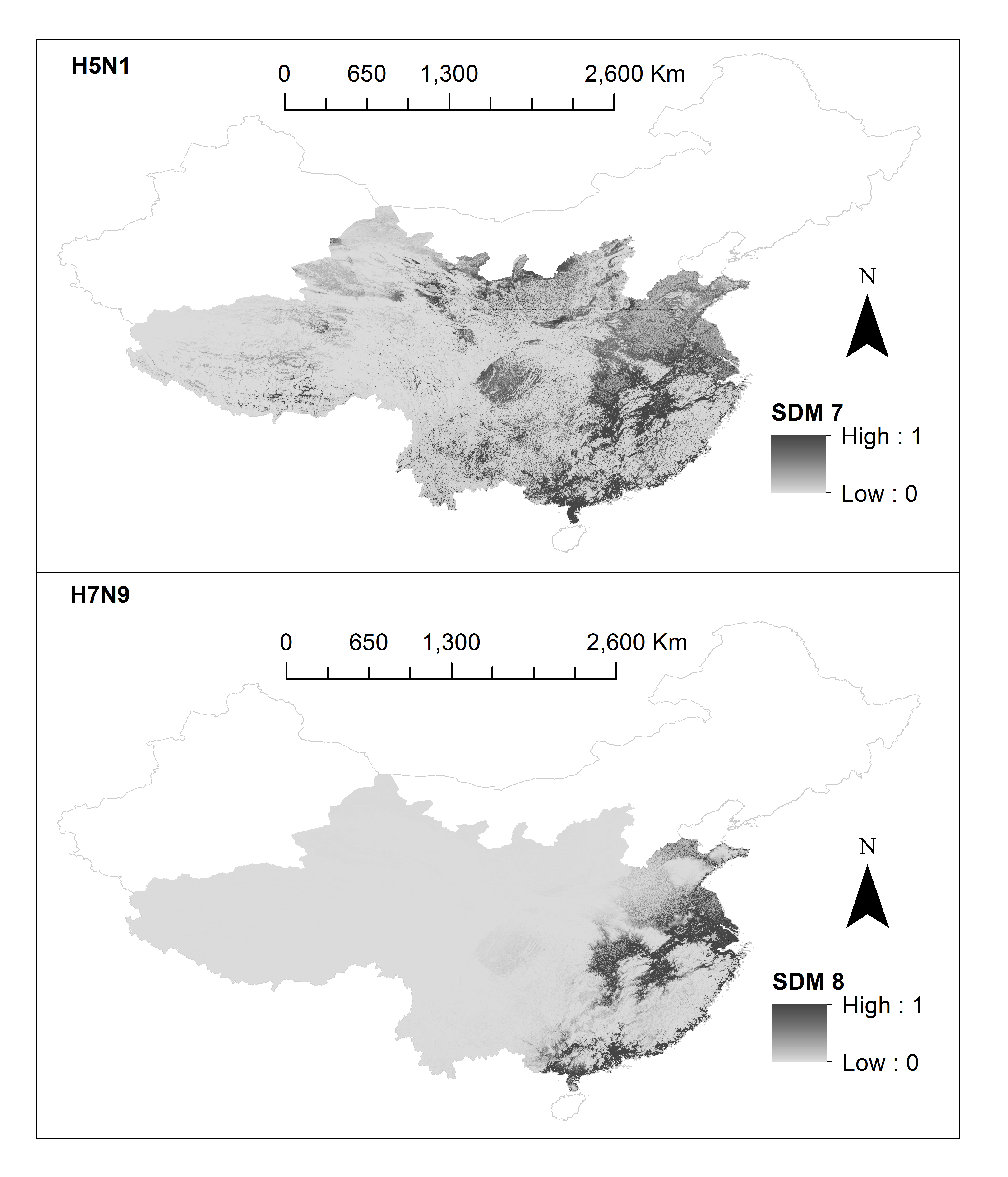

Supplement: S7 Fig — The first panel shows H5N1 (SDM 7) and the second panel shows H7N9 (SDM 8). Suitability values for each cell (approximately 1km2) are represented on a continuous scale of low (light grey) to high (dark grey). SDMs were built using Maxent software version 3.3.3k (available from https://www.cs.princeton.edu/~schapire/maxent/). SDMs were developed using environmental variables, created using data from: the WorldClim database (www.wordlclim.org), the Shuttle Radar Topography Mission (SRTM) 90m Digital Elevation Database v4.1 (www.cgiar-csi.org). Data sources used to obtain the case locations to build SDMs include: the Food and Agricultural Organization (FAO) (http://empres-i.fao.org/eipws3g/), the Chinese Ministry of Agriculture Avian Influenza Surveillance Reports (www.syj.moa.gov.cn), the World Organization of Animal Health (OIE) reports (www.oie.int). Base maps were obtained from the GADM database of Global Administrative Areas (http://www.gadm.org/). Maps were built using ArcMap 10.2. (TIF) [file pone.0174980.s007.tif]

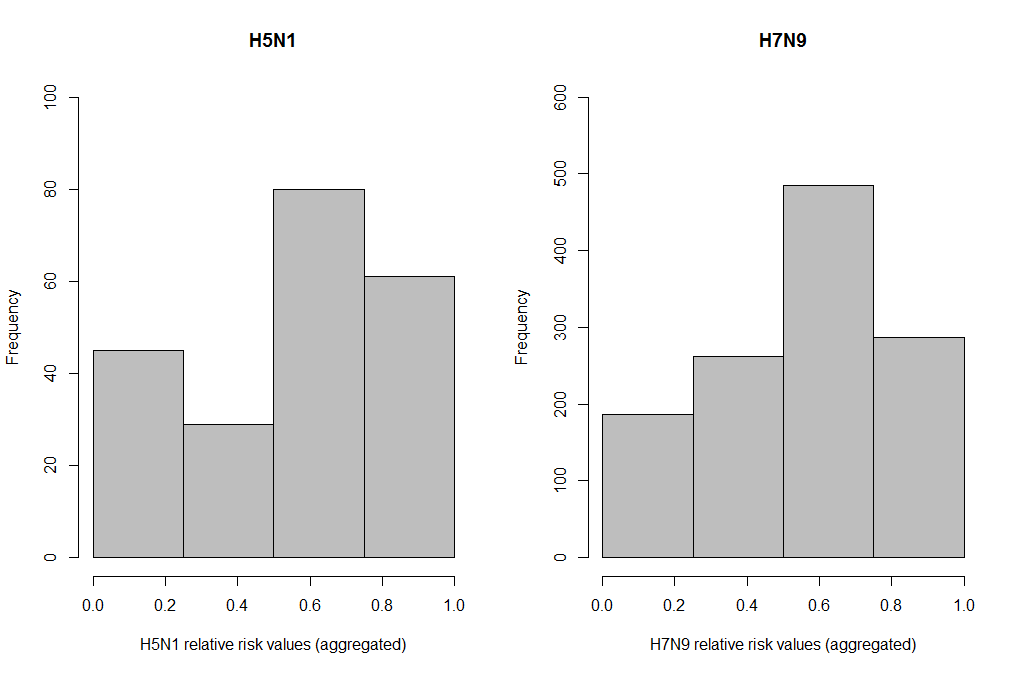

Supplement: S8 Fig — Number of all (unexact and exact) points per risk category (low 0.0–0.25; low-medium 0.25–0.50; medium-high 0.50–0.75; high 0.75–1.00). For each point, the maximum 1km cell relative risk value within approximately 5km radius was taken as the aggregated relative risk value. (TIF) [file pone.0174980.s008.tif]
