# Supplementary material for: Influenza A H5N1 and H7N9 in China: A spatial risk analysis
Source: PLoS One. 2017 Apr 4;12(4):e0174980. doi: 10.1371/journal.pone.0174980 (PMC5380336; doi:10.1371/journal.pone.0174980)
Supplement: S1 Table — (DOCX) [file pone.0174980.s009.docx]

Table S1. Summary of H5N1 Exact locations

|  | **Categories** | **Number** |
| --- | --- | --- |
| **Date of onset/**  **report** | 2004 | 17 |
|  | 2005 | 4 |
|  | 2006 | 6 |
|  | 2007 | 1 |
|  | 2008 | 4 |
|  | 2009 | 1 |
|  | 2011 | 1 |
|  | 2012 | 2 |
|  | 2014 | 13 |
|  | 2015 | 3 |
| **Animal host** | **Domestic poultry (total)** | **45** |
|  | *Chicken* | *13* |
|  | *Duck* | *2* |
|  | *Mixed (chicken/duck/ geese)* | *30* |
|  | Human | 1 |
|  | Tiger | 2 |
|  | Wild bird | 4 |
| **Location: description** | Farm | 3 |
|  | Lake | 4 |
|  | Market | 11 |
|  | Unspecified | 17 |
|  | Village | 15 |
|  | Zoo | 2 |
| **Total number of exact cases** |  | **52** |
