# Supplementary material for: Influenza A H5N1 and H7N9 in China: A spatial risk analysis
Source: PLoS One. 2017 Apr 4;12(4):e0174980. doi: 10.1371/journal.pone.0174980 (PMC5380336; doi:10.1371/journal.pone.0174980)
Supplement: S2 Table — (DOCX) [file pone.0174980.s010.docx]

Table S2. Summary of H7N9 Exact locations

|  | **Categories** | **Number** |
| --- | --- | --- |
| **Date of**  **onset/ report** | 2013 | 16 |
|  | 2014 | 21 |
|  | 2015 | 32 |
| **Animal host** | **Domestic poultry (total)** | **59** |
|  | *Chicken* | *37* |
|  | *Duck* | *6* |
|  | *Pigeon* | *2* |
|  | *Mixed (chicken/duck/ environmental)* | *4* |
|  | *Environment* | *7* |
|  | *Unspecified* | *3* |
|  | Human | 9 |
|  | Environment | 7 |
|  | Wild bird | 1 |
| **Location: description** | Market | 54 |
|  | Farm | 1 |
|  | Park | 1 |
|  | Slaughterhouse | 1 |
|  | Not specified | 12 |
| **Total number of exact cases** |  | **69** |
