# Supplementary material for: Influenza A H5N1 and H7N9 in China: A spatial risk analysis
Source: PLoS One. 2017 Apr 4;12(4):e0174980. doi: 10.1371/journal.pone.0174980 (PMC5380336; doi:10.1371/journal.pone.0174980)
Supplement: S3 Table — (DOCX) [file pone.0174980.s011.docx]

**S3** **Table. Summary of variables used in risk analysis**

| Variable | Coverage of original data | Format of original data | Interpretation of original data values | Resolution of original data | Data (reformatted) | Source |
| --- | --- | --- | --- | --- | --- | --- |
| Chicken density (extensive farms) | World | Raster | Birds per km^2^ | 1km | Continuous | **[1]** |
| Chicken density (intensive farms) | World | Raster | Birds per km^2^ | 1km | Continuous | **[1]** |
| Human population density | China | Raster | Average/ ambient human population distribution | 30 arc-seconds (~ 1km) | Continuous | **LandScan (2014)™** |
| SDM 3-4 | China | Raster | Probability of H5N1 or H7N9 presence | 30 arc-seconds (~ 1km) | Continuous | Created in this study |

1. Robinson TP, Wint GR, Conchedda G, Van Boeckel TP, Ercoli V, Palamara E, et al. (2014) Mapping the global distribution of livestock. PLoS One 9: e96084.
